# CD271 determines migratory properties of melanoma cells

**DOI:** 10.1038/s41598-017-10129-z

**Published:** 2017-08-29

**Authors:** Josefine Radke, Florian Roßner, Torben Redmer

**Affiliations:** 1 German Consortium for Translational Cancer Research (DKTK), German Cancer Research Center (DKFZ), Im Neuenheimer Feld 280, D-69120 Heidelberg, Germany; 2 Department of Neuropathology, Berlin, Germany; 3 Laboratory of Molecular Tumor Pathology, Berlin, Germany; 4 Institute of Pathology, Berlin, Germany; 5 Charité – Universitätsmedizin Berlin, Charitéplatz 1, D-10117 Berlin, Germany; 6 Berlin Institute of Health (BIH), Berlin, Germany

## Abstract

Melanoma cell expression of the nerve growth factor receptor CD271 is associated with stem-like properties. However, the contributing role of the receptor in melanoma cell migration is elusive. Here, we explored extracranial (skin, soft tissue, lymph node and liver, n = 13) and matched brain metastases (BM, n = 12) and observed a heterogeneous distribution of phenotypically distinct subsets of CD271^+^ cells. In addition, we observed that CD271 expression gradually rises along with melanoma progression and metastasis by exploration of publicly available expression data of nevi, primary melanoma (n = 31) and melanoma metastases (n = 54). Furthermore, we observed highest levels of CD271 in BM. Sub-clustering identified 99 genes differentially expressed among CD271^high^ and CD271^low^ (p < 0.05) BM-subgroups. Comparative analysis of subsets revealed increased ( ≥ 1.5fold, log2) expression of migration-associated genes and enrichment of CD271-responsible genes involved in DNA-repair and stemness. Live cell-imaging based scratch-wound assays of melanoma cells with stable knock-down of CD271 revealed a significantly reduced cell migration (3.9fold, p = 1.2E-04) and a reduced expression of FGF13, CSPG4, HMGA2 and AKT3 major candidate regulatory genes of melanoma cell migration. In summary, we provide new insights in melanoma cell migration and suggest that CD271 serves as a candidate regulator, sufficient to determine cellular properties of melanoma brain metastatic cells.

## Introduction

Distant metastasis is still the major obstacle to overcome in melanoma therapy, associated with poor prognosis and a ten-year survival rate of patients with distant metastases (stage IV) <10%^1^. Metastatic dissemination of primary tumors is an early event^2^ and the majority of patients exhibit regional or distant metastases by the time of diagnosis. Melanoma cells feature a high migratory phenotype^3^ facilitating the colonization of distant organs e.g. lung, liver, heart, peritoneum, small intestine, spleen and brain^4^. Despite this broad spectrum of possibly involved organs, brain metastases are very common, observed in 20–40% of melanoma patients. In addition, brain metastases are actually found *post mortem* in more than 75% of melanoma patients^5^. Moreover, multiple brain metastases (>5 intracerebral metastatic lesions) are observed in 5% of melanoma patients^6^ and may derive either from one founder clone or represent independent clones of different metastatic melanoma cells. Overall, the emergence of brain metastases is associated with poor prognosis due to limited therapeutic options. Stereotactic or whole-brain radiotherapy in combination with chemotherapy or immune-checkpoint inhibitors^7^ has recently gained increasing attention as meaningful therapeutic option for melanoma patients with brain metastases.

Migration and invasion of tumor cells are essential steps in the metastasis sequence^8^. Recently, the expression of nerve growth factor receptor CD271 was associated with increased incidence of melanoma brain metastases^9^ as well as metastases in lung, liver and kidney^10^. Furthermore, the BRAF^V600E^ mutation intrinsically confers a high migratory phenotype to melanoma cells^11^, blocked by the potent RAF-kinase inhibitor vemurafenib. Contrary, patients under vemurafenib therapy show a higher incidence for brain metastases as compared with patients who did not receive vemurafenib^12^. In addition, acquisition of melanoma cell resistance to vemurafenib as well as a higher tendency of brain metastasis was associated with expression of CD271^13, 14^. Hence, CD271 expression may prime melanoma cells intrinsically for extensive migration, metastasis and brain tropism. Apart from melanoma, other tumor entities bearing CD271^+^ cells^15^ also show comparable prevalence for brain metastasis, e.g. breast cancer (15–30%, reviewed in ref. 16). In glioblastoma, CD271^+^ cells represent a cellular sub-set highly capable of migrating and infiltrating the brain parenchyma^17^. However, it remains elusive whether CD271^+^ cells present a cell subpopulation prone to metastasize to the brain.

Here we explored the presence and distribution of CD271 expressing cells in primary melanoma as well as in extracranial, solitary and multiple brain metastases and elucidated the potential role of CD271 in melanoma brain tropism.

## Results

### Expression of CD271 discriminates melanoma progression stages and defines subsets of melanoma metastases

**Figure 1:**
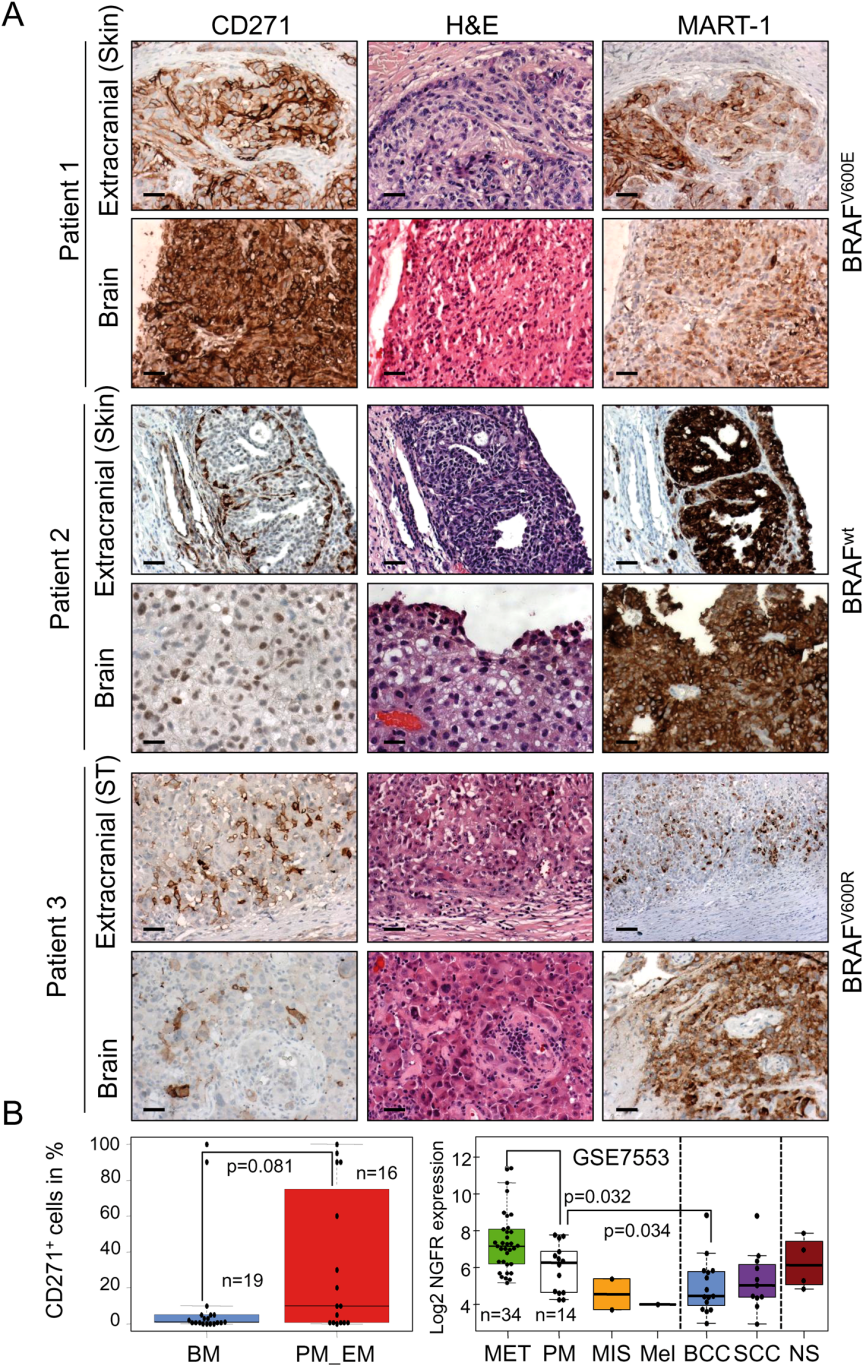
CD271 expressing cells are phenotypically distinct in extracranial and brain metastases. (**A**) Immunohistochemistry of three representative matched pairs of melanoma extracranial (EM) and corresponding brain metastases (BM, Patient 1 and 2), or soft tissue metastases (MET) and corresponding BM (Patient 3) for presence of CD271 and MART1 or hematoxylin/eosin (H&E). Scale bars indicate 50 µm. (**B**) Left panel: Box-plots show the number of CD271^+^ cells (%) of BM (n = 19), primary tumors (PM; n = 2) and EM (n = 14) as determined by counting of 50 visual fields at 200x magnification, p = 0.081. Right panel: Visualization of CD271/NGFR expression levels (log2) of skin cancer subtypes (GSE7553) including MET (n = 34), PM (n = 14), melanoma *in-situ* (MIS; n = 2), melanocytes (Mel.; n = 1), basal cell carcinoma (BCC; n = 15), squamous cell carcinoma (SCC; n = 11) and normal skin (NS). Significant difference in expression levels among MET/PM (p = 0.032) and PM/BCC (p = 0.034) is shown. Black filled circles indicate number of samples. If not stated otherwise, p-values were calculated by Wilcoxon rank-sum test.

Melanoma cells facilitate a high migratory phenotype^18^ capable of radial or vertical migration, reviewed in ref. 19. To explore whether CD271^+^ melanoma cells are prone to metastasize to the brain, we analyzed matched pairs of primary tumors (n = 2), extracranial (n = 13) and brain metastasis (n = 12) as well as unmatched brain (n = 7) and extracranial (n = 1) metastases of melanoma for expression of CD271, irrespective of the BRAF mutation status and therapeutic interventions (Supplementary information, SI; Table S1). We observed a median CD271 expression of 32% (range 0.5–100%) in extracranial and 11.9% (range 0.5–100%) in brain metastases. Hence, expression of CD271 was not significantly increased in either of these groups (Fig. 1A,B) and reflected strong inter-sample variations among melanoma metastases and primary tumors (Fig. 1B, left panel, p = 0.081; SI, Figures S1 and S2A,B). Highest CD271 expression (90%) was observed in a desmoplastic neurotrophic melanoma (DNM) and corresponding skin (n = 2) and brain metastases (n = 1), (Fig. 1A and SI, Figure S3A; Patient 1). In superficial spreading melanoma (SSM), we observed randomly distributed CD271^+^ cells within the tumor bulk or expression of CD271 at tumor edges and a wide-spread distribution in a soft tissue (ST) metastasis (Fig. 1A, Patients 2, 3). In healthy skin, CD271 expression was found in proliferating and non-proliferating (Ki67^+^/Ki67^−^) cells of the basal layer, and MART1^+^ or MITF^+^ melanocytes (SI, Figure S4A). Exploration of lymph node metastases not only revealed the presence of CD271^+^ tumor cells but also a prominent expression of CD271 in adjacent lymph follicles (LN) (SI, Figure S4B).

In a primary tumor of uveal melanoma (n = 1) we observed CD271 expression in the choroid cells as well as in spindle-shaped (Spin) but not epithelioid-appearing (Epi) tumor cells (SI, Figure S5A). In the associated liver and brain metastasis, we observed expression of CD271 in tumor cells (T) as well as in interstitial or stromal and vessel-adjacent cells (SI, Figures S5B,C).

**Figure 2:**
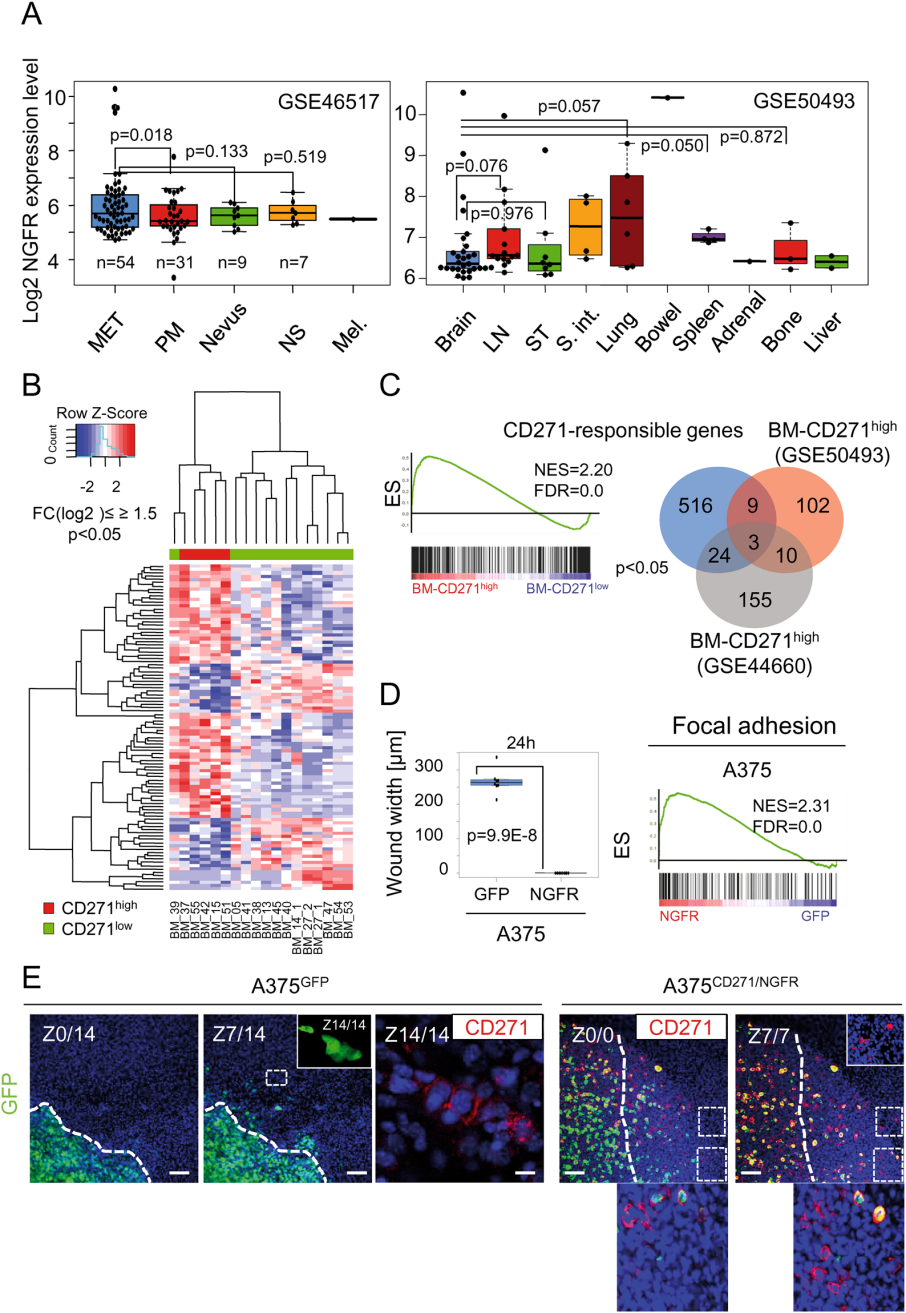
CD271 expression characterizes melanoma metastases. (**A**) Left panel: Visualization of CD271/NGFR expression levels (log2) at different melanoma progression stages (GSE46517): MET (n = 54), PM (n = 31), melanocytic nevus (Nevus; n = 9), NS (n = 7) and melanocytes (Mel.; n = 1). Right panel: CD271/NGFR expression levels (log2) of brain metastases (Brain; n = 29, GSE50493) as well as extracranial metastases, lymph node (LN; n = 12), soft tissue (ST; n = 10), small intestine (S. int.; n = 4), lung (Lung; n = 6), Bowel (n = 1), Spleen (n = 4), Adrenal (n = 1), Bone (n = 3), Liver (n = 2). (**B**) Supervised clustering of brain metastases with high expression of CD271/NGFR (red, patients 15, 37, 42, 51 and 55) and very low expression (green, n = 13) of 99 genes, among them 62 genes with increased (≥1.5fold, log2; p < 0.05) and 37 genes with decreased (≤1.5fold, log2; p < 0.05) expression. (**C**) Left and right panel: Gene-set enrichment analysis (GSEA) of CD271^high^ (BM-CD271^high^) and CD271^low^ (BM-CD271^low^) brain metastases (study GSE50493) shows enrichment of CD271-responsive genes (n = 156; NES = normalized enrichment score; FDR = false discovery rate). Venn-diagram depicts the number of CD271-responsible genes, distinctly or commonly up-regulated in CD271^high^ brain metastases in two independent studies (GSE50493, GSE44660; FC_log2_ ≥ 1.1, p ≤ 0.05). (**D**) Left panel: Representation of the migratory capacity of A375^GFP^ and A375^CD271/NGFR^ cells indicated by the wound width [µm], 24 hours after wounding. Right panel: Enrichment of genes involved in focal adhesion formation (Broad institute’s MSigDB) in A375^CD271/NGFR^ cells as determined by GSEA (**E**) Confocal microscopy-based serial imaging of mouse brain slices, 48 h after injection of 10 000 A375^GFP^ or A375^CD271/NGFR^ cells. Left panels: GFP expression of image sections, Z0/0, Z7/14, Z14/14 (inset) as well as CD271 expression (red) for Z14/14 of slices injected with A375^GFP^ are shown. Right panels: image sections Z0/7and Z7/7 of slices injected with A375^CD271/NGFR^ are shown. Areas depicting migrating CD271^+^ cells are magnified. DAPI served as nuclear stain, scale bars indicate 50 µm. White dotted lines indicate initial tumor borders/non-migrated cells.

Further, we explored publicly available expression data (GSE7553^20^) of melanoma metastases (MET, n = 34), primary melanoma (PM, n = 14), melanoma *in situ* (MIS, n = 2), melanocytes (Mel, n = 1), basal cell carcinoma (BCC, n = 15), squamous cell carcinoma (SCC, n = 11) and normal skin (NS, n = 4). We observed highest CD271 expression in MET, as compared to PM (p = 0.032) which in turn show higher CD271 levels as compared to BCC (p = 0.034) and SCC (not significant, NS) (Fig. 1B, right panel). In addition, we observed a high variance of CD271 expression levels among primary melanoma subtypes: Acral, SSM, Nodular, Desmoplastic, LMM and spindle cell melanoma (GSE15605^21^), (SI, Figure S6A). We next asked for levels of CD271 expression along stages of melanoma evolution and progression (GSE46517^22^). We observed that expression of CD271 increased with melanoma progression and attained significantly (p = 0.018) highest levels in MET (n = 54) as compared to PM, n = 31 (Fig. 2A, left panel), which is in line with a previous observation^23^. Together, our findings are underpinned by public data and demonstrate the existence of subsets of melanoma metastases with high expression of CD271. This was further evaluated with expression data of metastases of different metastatic sites (GSE50493). We observed a subset of BM (n = 5 of 29, 17.2%) with strong CD271 expression (Fig. 2A, right panel). Next, we asked whether a high level of CD271 indeed determines the character of these CD271^high^ tumors. A comparative analysis of expression data of CD271^high^ BM (BM-CD271^high^) or low (CD271^low^) CD271 expression led to identification of 2834 differentially regulated genes (p ≤ 0.05). Among them we identified 62 genes with increased ( ≥ 1.5fold, log2; p ≤ 0.05) and 37 genes with decreased ( ≤ 1.5fold, log2; p ≤ 0.05) expression in the CD271^high^ subset as compared to CD271^low^ tumors (Fig. 2B). Tumors of the CD271^high^ subset showed a ~5fold higher CD271 expression (p = 0.002) as compared to CD271^low^ brain tumors (n = 13), (SI, Figure S6B-C). Among the top regulated genes we found those associated with cell migration e.g. NRCAM^24^ (neural cell adhesion molecule), TWIST1^25^ or LOX^26^ (lysyl oxidase) (Fig. 2B). In addition, gene-set enrichment analysis (GSEA) of both subsets of brain tumors demonstrated that CD271 expression is associated with enrichment of CD271-responsible genes^27^ (NES = 2.20), (Fig. 2C). Next, we asked for CD271-responsible genes potentially up-regulated in the CD271^high^ subgroups of brain metastases (BM) in independent data sets (GSE50493, GSE44660). We found CD271-responsible genes up-regulated in either of the two groups of CD271^high^ BM (Fig. 2C, right panel). In addition, we found commonly regulated genes associated with metastasis; TUBB2B^28^ and PMEPA1^29^, besides NGFR. Further exploration of genes differentially regulated among the CD271^high^ and CD271^low^ BM subsets revealed commonly up-regulated (n = 10) and down-regulated (n = 5) genes. Among genes with significantly increased expression in the CD271^high^ subsets were mediators of tumor cell migration e.g. AXL^30^ and members of the lysyl-oxidase family LOX^26^ and LOXL2^31^ as well as the less characterized SERTAD4 (SERTA Domain-Containing Protein 4), (SI, Figure S6D-E). In addition, we found increased expression of the receptor-tyrosine kinase c-MET in the CD271^low^ groups only (SI, Figure S6E). Additional GSEA of CD271^high^ and CD271^low^ subsets identified a global enrichment of genes associated with DNA-repair (NES = 2.05), a neural-crest cell stem-like (NCSC) state (NES = 1.89) or formation of cell projections, a prerequisite for cell migration and a brain metastatic phenotype in the subset of CD271^high^ BM (GSE50493), (SI, Figure S7A and S7B, left panel). Furthermore, we observed enrichment of genes associated with a EMT (epithelial to mesenchymal transition) cellular phenotype and CD271-dependent response in CD271^high^ BM of GSE44660 (SI, Figure S7B). Together these data suggest that CD271 serves as a subclassifier of melanoma brain metastases.

Tumor cell metastasis relies on the formation of focal adhesive contacts and the migratory capacity^8^. We previously observed a high migratory phenotype of A375 melanoma cells which was strongly enhanced (~15fold, p = 9.9E-8) by over expression of CD271 (A375^CD271/NGFR^ cells)^27^, (Fig. 2D, left panel). We observed that genes associated with focal adhesion as well as lamellopodia structures are strongly enriched in A375^CD271/NGFR^ cells (Fig. 2D, right panel and SI, Figure S7C) as compared to A375^GFP^ cells. In addition, we followed migration of A375^GFP^ cells in mouse brain slice cultures by sequential confocal imaging to exemplify cell migration in a three-dimensional system (Movie files S1-S2). A375^GFP^ cells showed proliferation within the slice as monitored by immunofluorescence microscopy (SI, Figure S8A, upper panels) and the tracking of A375^GFP^ cells revealed their extensive migratory capacity (Fig. 2E, left panels and SI, Figure S9A-B) and showed the presence and distribution of migrating CD271^+^ cells. We tracked migration of A375^GFP^ cells with endogenous CD271 expression as soon as 48 hours post injection, regarding our previous *in vitro* data^27^. Although the quantification of cells migrating within slices is difficult, we attempted to determine the number of cells capable of long distance migration. We measured the distance of the lower edge of the initial tumor to the most distantly migrated cells and observed that ~0.2–0.4% of cells reached a distance of 267–534 µm, among them were CD271^+^ cells (Fig. 2E; SI, Figure S9A). However, to ascertain whether a increased expression of CD271 is favorable for melanoma cell migration, A375^CD271/NGFR^ cells which show a high constitutive and stable cell surface expression of CD271 (SI, Figure S7C, left panels) not affected by phenotype switching processes, were injected in parallel to control cells (A375^GFP^). Tracking of A375^CD271/NGFR^ cells demonstrated that CD271^+^ cells are indeed capable of intracerebral migration (Fig. 2E, right panels; SI, S10A-B and Movie file S3) and proliferation (SI, Figure S8A, lower panels) following adaption to environmental cues of the brain tissue. In line is the finding of migrating/infiltrating CD271^+^ melanoma cells in melanoma brain metastases (SI, Figure S8B, Patient 4).

### CD271 knock-down impairs melanoma cell migration

**Figure 3:**
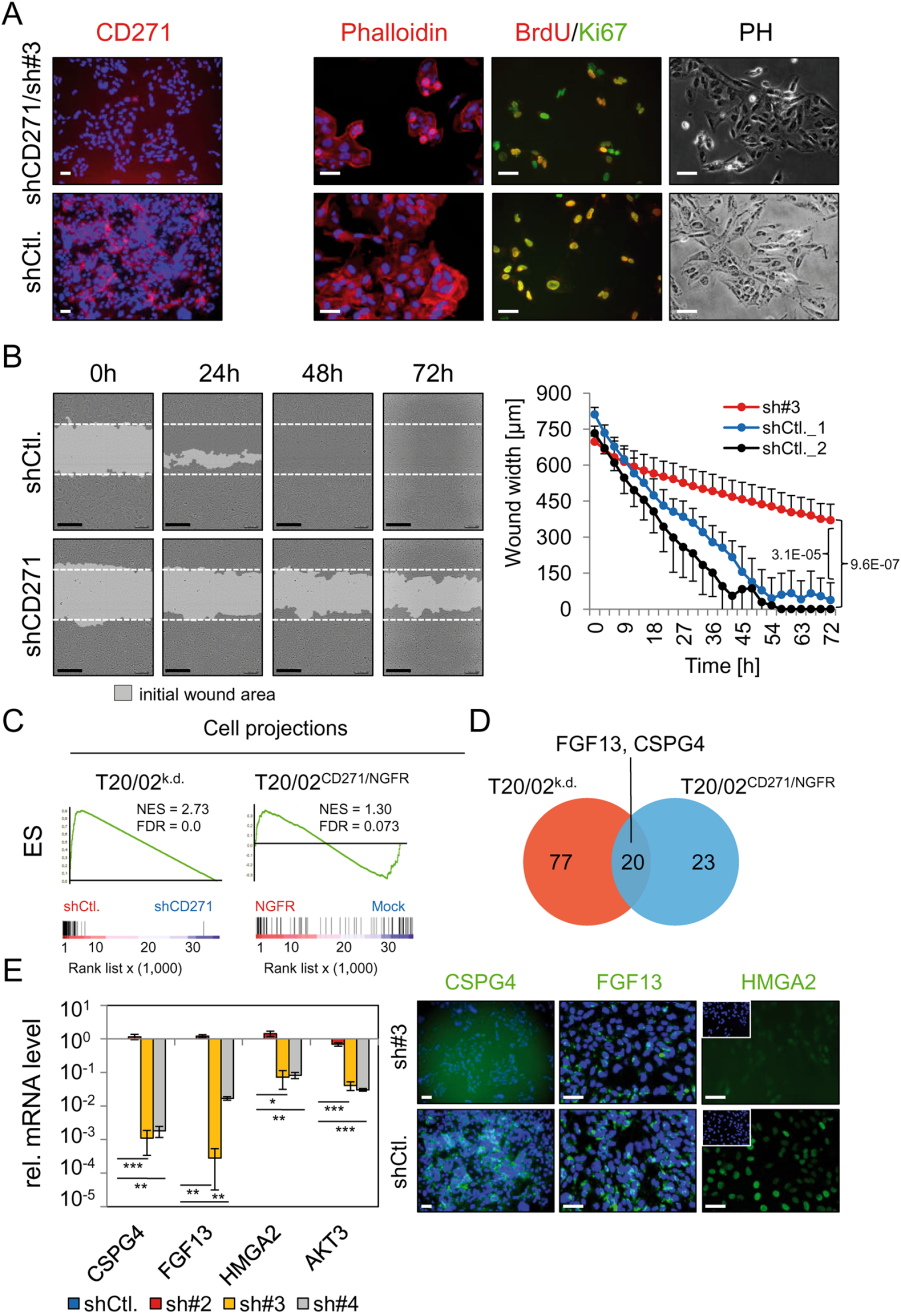
CD271 controls expression of mediators of cell migration. (**A**) Left panels: Absence of CD271 in melanoma cells (T20/02) stably transfected with a CD271-targeting shRNA (shCD271/sh#3) but high expression in control cells (shCtl.) as determined by immunofluorescence and a directly labeled (PE, red) CD271 antibody. Right panels: A changed morphology of CD271 knock-down cells is indicated by phalloidin (red) and bright field images (PH), as well as a decreased number of BrdU^+^ (red) but not Ki67^+^ (green) cells as compared to shCtl. cells. (**B**) Left panel: Live cell-imaging based scratch-wound assay of cells described in (A) at 0, 24, 48 and 72 hours after wounding. Initially, 30 000 cells/well (96-well plate) were seeded, reproducible wounds were scratched using the wound maker tool (Essen bioscience). Scale bars indicate 200 µm, the initial wound is indicated by white dotted lines. Right panel: Quantification of the scratch-wound assay, the wound width [µm] was determined every 3 hours. Shown are mean values ± sdv of n = 8 replicates, p = 3.1E-05 (shCtl.1 vs. sh#3) or 9.6E-07 (shCtl.2 vs. sh#3), ttest. (**C**) Left and right panel: CD271-responsible regulation of genes associated with formation of cell projections as determined by GSEA of T20/02 cells engineered for stable down-regulation (T20/02^k.d.)^ or over expression (T20/02^CD271/NGFR^) of CD271. (**D**) Representation of genes found either up-regulated or down-regulated by knock-down or over expression, respectively. A common set of 20 genes followed a CD271-responsible regulation among them FGF13 and CSPG4. (**E**) Left panel: Determination of expression levels of potential migration-associated genes CSPG4, FGF13, HMGA2 and AKT3 by qPCR of T20/02 cells either stably transfected with control shRNA (shCtl) or effective CD271-targeting shRNAs (sh#3, sh#4; sh#2, not effective). Relative expression levels are shown as mean values ± sdv of biological triplicates, ***p ≤ 0.001, **p ≤ 0.01, *p ≤ 0.05, ttest. Right panel: Immunofluorescence microscopy for levels of CSPG4, FGF13 and HMGA2 in shCtl. and CD271 knock-down cells (sh#3). DAPI served as nuclear stain, scale bars indicate 50 µm.

To further unravel the role of CD271 in melanoma cell migration we explored the migratory capacity of a patient tumor-derived cell strain^32^ following stable shRNA mediated knock-down of CD271. The efficient knock-down of CD271 with a validated shRNA (sh#3) (Fig. 3A, left panels) induced a strong change in morphology, without effecting the overall number of Ki67^+^ cells but decreased the number of BrdU^+^ cells (59.7 ± 8.5%, shCtl. vs. 34.3 ± 15.9%, shCD271/sh#3; p = 0.077, ttest), (Fig. 3A, right panels and SI, Figures S11A-B), therefore impaired proliferation. Next, we ascertained whether cell migration was affected by loss of CD271 expression in a live cell-imaging scratch-wound assay. Tracking migration of CD271 knock-down cells by imaging of the scratch wound every 3 hours (72 hours in total) revealed a strong decrease (1.3fold, p = 6.6E-03/3.9fold, p = 1.2E-04 or 9.8fold, p = 3.1E-05 at 24/48 or 72 hours) in the efficacy of wound closure as early as 24h, as compared to control (shCtl.) or GFP expressing cells (Fig. 3B, left panel and Movie files S4-S6). Confocal microscopy of mouse brain slices 5 days post injection of 5 000 cells of each (shCD271, sh#4 and control, shCtl.) revealed widespread migration of CD271^+^ control cells, exhibiting both radially and vertically migration (SI, Figure S12A and Movie file S6). In contrast, the knock-down cells, which we monitored via GFP expression showed no or only limited proliferation and migration (SI, Figures S12B-C and Movie file S8), underpinning the *in vitro* findings.

We next asked for the CD271-responsible regulation of factors potentially involved in cell migration like those associated with formation of cell projections. To this end, we explored our expression data sets of melanoma cells with knock-down (GSE52456) or over expression (GSE78155) of CD271 by GSEA and observed a depletion or enrichment of genes associated with formation of cell projections (Fig. 3C). The integrative analysis of both gene sets revealed a subset of directly CD271-responding genes (n = 20), known to be involved in cell migration or metastasis e.g. fibroblast growth factor 13 (FGF13)^33^, chondroitin sulfate proteoglycan 4 (CSPG4)^34^ (Fig. 3D). In addition, our screen identified other potential mediators of melanoma cell migration e.g. the high mobility group AT-hook 2 (HMGA2)^35^ transcription factor or AKT3^36^. We verified that expression of CD271 is crucial to maintain the levels of all four genes in melanoma cells stably expressing CD271-targeting shRNAs (sh#3/sh#4). qPCR of CD271 knock-down cells showed decreased levels of CSPG4 (p = 8.3E-04/8.1E-05), FGF13 (p = 2.1E-03/2.2E-04), HMGA2 (p = 1.8E-02/1.4E-04) and AKT3 (p = 6.5E-04/4.9E-06), (Fig. 3E, left panel). We further confirmed the loss of CSPG4, FGF13 and HMGA2 by immunofluorescence microscopy (Fig. 3E, right panel). Next, we ascertained the potential significance of these factors in melanoma migration and metastasis. To this end, we explored publicly available expression data of primary melanoma and extracranial metastases (GSE46517, GSE7553, GSE8401) for levels of HMGA2. We observed that HMGA2 was predominantly expressed in melanoma metastases with a significant difference in two out of three studies (p = 3.6E-06, p = 0.301 and p = 2.4E-05), (SI, Figures S13A, S14B). Since we observed a clear down-regulation of HMGA2 in CD271 knock-down cells, we asked for genes involved in melanoma migration and metastasis supposedly driven by a CD271/HMGA2 axis. We initially identified 1337 (FC_log2_ ≤ 0.5, p ≤ 0.05) potential HMGA2-targets by exploration of expression profiling data of the breast cancer cell line MDA-MB-231 with HMGA2 knock-down (GSE43741^37^). We then employed expression data of our previous study (GSE52456). The integrative analysis of both sets of targets identified 130 genes, commonly down-regulated upon knock-down of CD271 or HMGA2 (SI, Table S3). Among these potential CD271/HMGA2 targets were migration and metastasis-associated genes e.g. SEMA3A, ST3GAL1^38^, LOX^26^ and PDLIM1^39^. Further analyses demonstrated a significantly increased level of SEMA3A and ST3GAL1 or decreased level of PDLIM1 in MET (SI, Figure S13B, S14A-B). Next, we ascertained the dependence of HMGA2, PDLIM1 and SEMA3A expression of CD271. We ranked MET regarding their CD271 levels and observed a CD271-dependency of SEMA3A but not HMGA2 or PDLIM1 expression in CD271^high^ metastases (SI, Figure S14A-B). Further, we evaluated CSPG4 and FGF13 expression in MET and observed a non-significant or only partially significant difference in expression of CSPG4 or FGF13 among primary and metastatic melanoma (SI, Figures S13D, left panels; S13E and S14A), respectively. Although CSPG4 and CD271 are co-expressed in a subset of melanoma cells of MeWo^Par^ (parental MeWo) and T20/02 (SI, Figure S13D, right panels), their role in cell migration and metastasis might be different.

Activation of the PI3K/AKT pathway has been recognized as a potent driver of melanoma progression and metastasis, including metastasis to the brain^36, 40, 41^. We asked whether the decrease of AKT3 expression in CD271 knock-down cells affected the overall activation of this pathway. We observed a reduced level of activated AKT, phosphorylated at S473 (p-AKT^S473^) in CD271 knock-down cells but no changes in the levels of activated extracellular signal-regulated kinases 1/2 (p-ERK1/2), (SI, Figure S13F, left panels). In addition, sorting of melanoma cells for CD271 demonstrated a predominance of activated PI3K/AKT signaling in CD271^+^ cells (SI, Figure S13F, right panels).

Together, these data suggest that CD271 may control migration and metastasis mechanisms supposedly via a network of mediators e.g. HMGA2 or FGF13 or AKT- signaling (SI, Figure S14C).

### Inspection of multiple brain metastases reveal phenotypical concordance

The mechanisms underlying melanoma metastasis to the brain are still poorly understood. Therefore the identification of key players determining brain metastases is mandatory.

**Figure 4:**
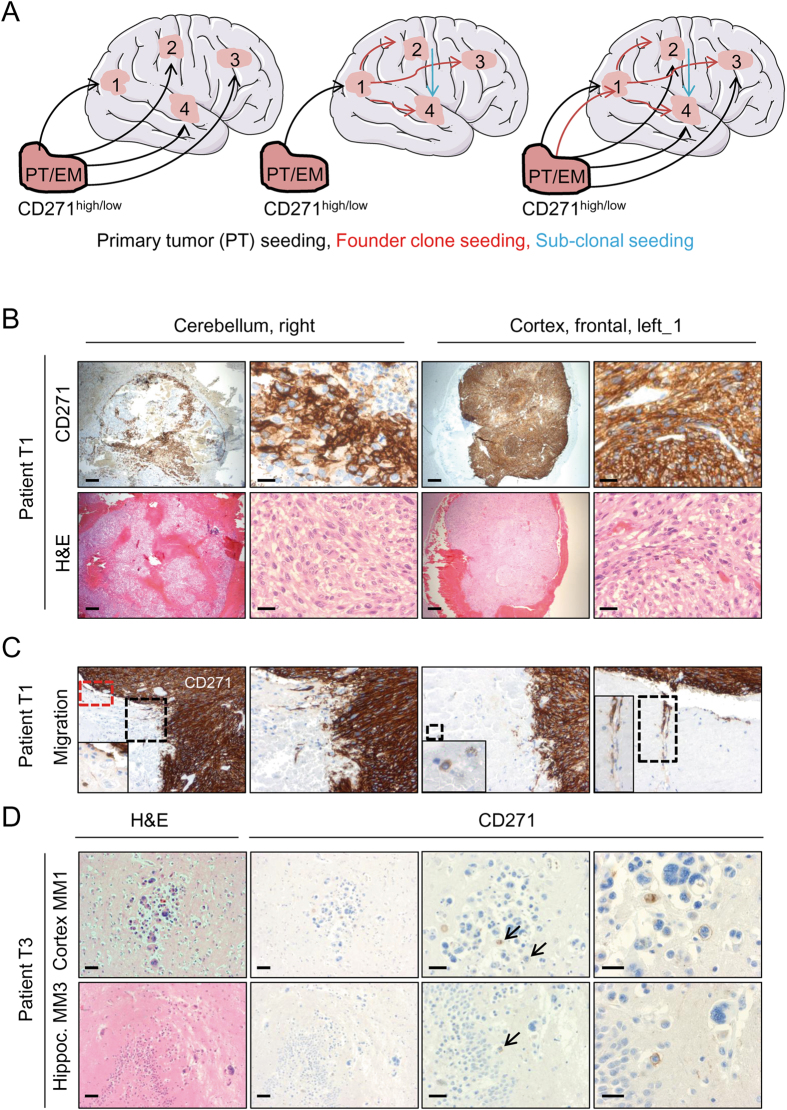
Exploration of multiple brain metastases reveal a phenotypical concordance of CD271 expression. (**A**) Schematic representation of plausible mechanisms of primary tumor (PM) or extracranial metastasis (EM) seeding to the brain suggesting either a multiple or single seeding of PM/EM or a combination of both leading to high phenotypic heterogeneity (left and right scheme) or phenotypic homogeneity (center scheme). Brain schemes were obtained from Servier (http://servier.com/Powerpoint-image-bank) as per Servier’s usage license (https://creativecommons.org/licenses/by/3.0/legalcode) and modified. (**B**) Immunohistochemistry of autopsied multiple brain metastases. In patient T1, all tumors (n = 3) located in the cerebellum and frontal cortex showed strong expression of CD271. (**C**) Areas indicating CD271^+^ cell migration /invasion into adjacent brain parenchyma of tumors described in (B), are shown. Insets show distant CD271^+^ cells. (**D**) Exploration of brain micrometastases located in the hippocampus and cortex of patient T3 for presence of CD271^+^ cells (black arrows).

In principle, several hypothetical scenarios for the seeding of primary tumors (PT) or extracranial metastases (EM) are plausible. Multiple seeding of CD271^high^ or CD271^low^ tumor cells should lead to intratumor heterogeneity of brain metastases (Fig. 4A, left scheme). The single seeding of PT/EM and establishment of a founder clone, derived from brain metastatic cells would increase the likelihood for the formation of multiple brain metastases with comparable phenotypes. The latter is given by seeding of the founder clone (Fig. 4A, center scheme). In another scenario, seeding of a founder clone and PT/EM again would lead to increased phenotypic heterogeneity among brain metastases (Fig. 4A, right scheme). Related to these potential mechanisms, we asked for the occurrence and dissemination of CD271^+^ cells among autopsied tumor samples of patients with multiple (n ≥ 2) brain metastases. Again, we found strong inter-patient heterogeneity of CD271 expression. Nevertheless, multiple brain metastases derived from one patient (T1) revealed a homogeneous distribution of CD271^+^ cells accompanied by a high expression of S100 but absence of MART1 (Fig. 4B and SI, Figure S15A). The relationship of CD271, S100 and MART1 was found in additional brain metastases of patient T2 (SI, Figure S15B). CD271 expression of tumors was distinct from neurons residing in the brain parenchyma. In addition, we observed infiltration of the adjacent brain parenchyma by migrating CD271^+^ cells (Fig. 4C), clearly suggesting that CD271^+^ cells are capable of seeding within the brain and potentially associated with the formation of multiple brain metastases. Next, we ascertained the presence of CD271^+^ cells in brain micrometastases of autopsied brain tissue samples (cerebellum, hippocampus, cortex) of a patient with additional metastases in liver, lung and spleen (Patient T3; SI, Table S2). We found CD271^+^ cells albeit with low frequency, in four out of six micrometastases of different regions, (Fig. 4D and SI, Figure S16A).

The dissemination of melanoma cells via the blood stream is not the only mechanism of distant metastasis. Besides this passive process, melanoma cells actively migrate along vessels by vessel co-option^42^. In brain metastases, we observed CD271^+^ cells adjacent to blood vessels as well as a clear, distinct staining of endothelial (CD31) and melanoma cells (CD271), (SI, Figure S16B). In addition, we very rarely observed CD271^+^ supposedly melanoma cells in a blood vessel (SI, Figure S16C) as well as a potential blood-vessel directed migration of CD271^+^ cells (SI, Figure S16D).

### Expression of the microphthalmia-associated transcription factor (MITF) is down-regulated in CD271^high^ brain metastases

**Figure 5:**
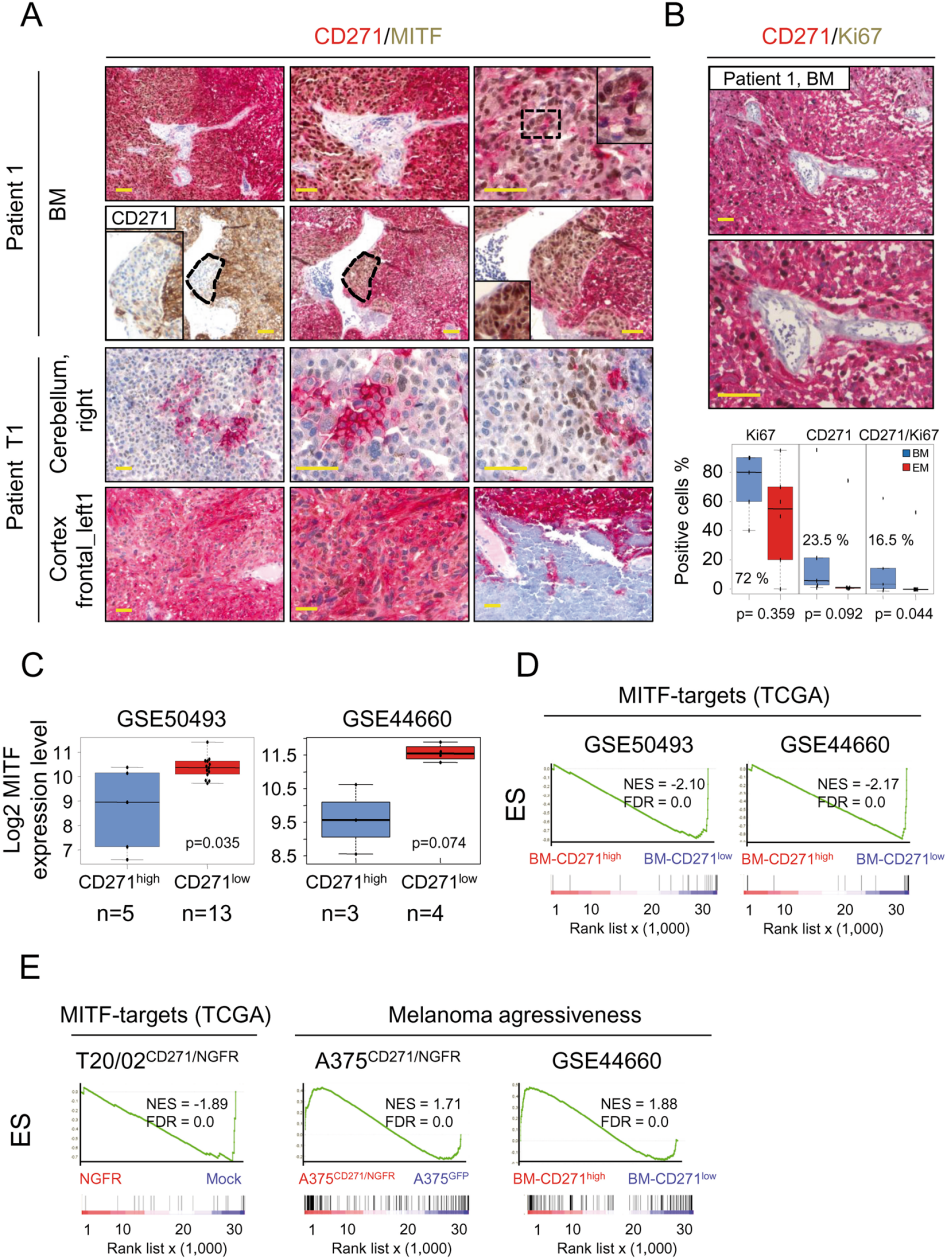
CD271 ^high^ brain metastases lack expression of MITF. (**A**) Co-immunohistochemistry of a solitary BM of patient 1 (two different tumor areas) as well as multiple BM of patient T1 for CD271 (membrane, red) and MITF (nuclear, brown). (**B**) Upper and center panels: Staining of patient 1 BM for CD271 and Ki67. Lower panel, quantification of Ki67^+^, CD271^+^ and double positive cells of BM (n = 5) and EM (n = 6). (**C**) MITF expression levels (log2) of CD271^high^ and CD271^low^ BM of two independent studies (GSE50493, GSE44660) are shown. (**D**,**E**) GSEA of BM subsets analyzed in (C) and T20/02 cells with over expression of CD271 (T20/02^CD271/NGFR^) for enrichment of MITF-target genes as provided by TCGA^43^, compared to untransfected cells (Mock) is shown. In addition, GSEA revealed enrichment of melanoma aggressiveness-associated genes in A375 cells (CD271/NGFR vs. GFP) and BM. In (B) and (C) p-values were calculated by Wilcoxon rank-sum test.

Low expression of MITF has been associated with a more aggressive, stem like and tumor-initiating phenotype of melanoma cells^43, 44^. We explored solitary (Patient 1) and multiple (Patient T1) melanoma BM with a CD271^high^ phenotype for expression of MITF by co-immunohistochemical staining. Basically, we observed a mutually exclusive expression pattern of CD271 and MITF and in addition a transition-state where expression of both markers was not clearly separated (Fig. 5A; Patient 1, BM). In multiple BM, MITF expression was not detected in areas strongly positive for CD271 (cortical metastasis). However, MITF was expressed in CD271 negative tumor areas adjacent to normal tissue in the cerebellar metastasis (Fig. 5A; Patient T1). Next, we determined the proportion of proliferating, Ki67^+^ cells in BM (n = 5) and EM (n = 6) and observed no correlation between CD271 expression and the proliferative state. Moreover, the non-proliferating CD271 expressing cells most likely constitute the stem-like subset, responsible for tumor progression as suggested previously^44^. In fact, a minority (~7% on average) of CD271^+^ cells in BM was in a non-proliferative state (Fig. 5B, lower panel). To further validate the inverse correlation of CD271 and MITF expression, we evaluated MITF expression levels and performed GSEA of CD271^high^ and CD271^low^ BM (GSE50493, GSE44660). Indeed, we observed a higher expression of MITF in CD271^low^ BM as well as an enrichment of MITF target genes^43^ (Fig. 5C-D). Furthermore, we found MITF target genes depleted in cells with forced expression of CD271 (NGFR) as well as a more aggressive phenotype of CD271^high^ A375 cells (A375^CD271/NGFR^) and BM (CD271^high^) by GSEA (Fig. 5E). In summary, these data suggest that the subset of CD271^+^ cells and brain metastases correlate with a MITF^low^ and more aggressive phenotype.

## Discussion

Melanoma cell spreading to distant organs is still a challenge in melanoma therapy and associated with poor survival. Especially metastasis to the brain is frequently observed in melanoma patients and most likely presents a homing process of melanoma cells governed by specific cytokines or growth factors supplied by the brain tissue^45^. However, mechanisms determining the brain metastatic capacity of melanoma cells are still poorly understood. Recently, we demonstrated that expression of the nerve growth factor receptor CD271 determines melanoma cell properties and discriminates PM and MET by regulation of CD271-responsive genes^27^. Encouraged by the previous finding of increased expression of CD271 in BM as compared to EM^9^ we sought to evaluate as to whether the expression of CD271 and responsible genes indeed promote metastasis to the brain. Our initial analysis of brain and extracranial metastases showed a heterogeneous expression pattern of CD271 with a subset of CD271^high^ BM. This was in line with publicly available expression data of melanoma brain metastases. Exploration of public data of BM of two independent studies demonstrated that expression of CD271 was sufficient to define a BM-subset exhibiting distinct features e.g. enrichment of CD271-responsible genes including those involved in DNA-repair, a stem-like phenotype or genesis of cell projections. Regarding the pronounced plasticity of melanoma cells^32, 46^, a high occurrence of CD271^+^ cells in melanoma BM may suggest that certain cellular phenotypes are possibly maintained in dependence of the prevailing microenvironment of tumors, epigenetic cues^47^ or stress e.g. induced by therapeutic interventions^48^ and may confer increased cell survival^49^. Comparison of both CD271^high^ subsets revealed commonly regulated genes, associated with migration and metastasis. Since the number of BM of study GSE44660 was comparably low, we assumed this to be responsible for the low overlap of both studies. However, verification of individual genes common to both sets of CD271^high^ BM showed a significant difference among CD271^high^ and CD271^low^ BM, suggesting CD271 to determine properties of a BM subset.

Remarkably, the BRAF^V600E^ mutation intrinsically confers a high migratory phenotype to melanoma cells, which is blocked by the RAF-kinase inhibitor vemurafenib^11^. Nevertheless, patients under vemurafenib therapy show a higher incidence of brain metastases^12^ which is properly reasoned by the high brain metastatic potential of melanoma cells resistant to vemurafenib^14^. The latter cells show increased levels of CD271 which in turn is up-regulated via activation of the NFkB signaling pathway in BRAF-mutated melanoma cells^13^, most likely conferring increased migration. We observed a clear migratory phenotype of A375^GFP^ cells *in vitro* and *ex vivo* (semi-*in vivo*) in organotypic brain slices. In fact, we observed CD271^+^ cells showing long-distance migration through the slice. We attempted to exemplary quantify the number of A375^GFP^ cells showing a long-distance migration in the organotypic brain slice by counting of migrated cells. Nevertheless, an absolute quantification of migrating A375^GFP^ cells is hampered by the lack of detailed insight into rates of proliferation and apoptosis within the slice.

CD271 expression - like other melanoma markers - underlies phenotype switching responsible for fluctuations of CD271 expression^44, 50^. We demonstrated that A375 cells with stable over expression of CD271 are indeed capable of migration in brain slices. This may suggest that a CD271^+^ phenotype of brain metastases is not necessarily acquired following colonization but rather predetermined by migrating CD271^+^ cells. This is underpinned by our previous observation that over expression of CD271 enhanced migration of A375 cells *in vitro* ^27^, suggesting an additional effect of CD271, potentially superior to the effects driven by oncogenic BRAF. However, mechanisms of CD271-dependent cell migration processes remain elusive and further studies are needed to further explore this issue.

Exploration of CD271 and CD31 expression in solitary and multiple BM revealed that CD271^+^ cells are localized around CD31^+^ vessels. Although we also observed CD271^+^ cells inside a blood vessel, this observation was extremely rare but may suggest a hematogenous dissemination of CD271^+^ cells into the brain as well as their migration along blood vessels^51^ (reviewed in ref. 42). Additionally, lymphatic spread of melanoma cells is of major prognostic relevance and the presence of CD271^+^ cells in lymph node metastases suggest a dual mechanism of dissemination.

Mechanisms involved in melanoma cell metastasis have been described previously, e.g. involving the cytokine receptor CXCR4^52, 53^. However, this issue seems to be more complex and is determined by multiple factors, e.g. melanoma cell plasticity. We previously identified a CD271-dependend regulation of FGF13, a special type of non-secreted fibroblast growth factors, suggested to regulate neuronal microtubule assembly and stabilization^33^, migration, and chemo-resistance^54^. Together, this might suggest a CD271/FGF13 axis as yet another regulatory mechanism of melanoma cell migration. In addition, we found other factors known to be involved in cell migration, regulated in a CD271-dependent manner, e.g. CSPG4^34, 55^ and HMGA2^35^, strongly indicating that CD271 expression is a prerequisite for proper melanoma cell migration. To further unravel a potential connection of CD271 and cell migration, we sought to identify genes responsible to HMGA2 and CD271. Since expression data of HMGA2 knock-down melanoma cells were not available, we utilized expression data of a breast cancer cell line (MDA-MB-231) with HMGA2 knock-down^37^ and identified HMGA2-responsible genes. Comparison of HMGA2- and CD271-responsible genes revealed a commonly regulated set of 130 genes, suggesting the indirect regulation of HMGA2 targets via CD271 and the potential existence of a HMGA2/CD271 network.

The proteoglycan CSPG4 is associated with cell migration or proliferation by regulation of small GTPases or fine-tuning of growth factor receptor signaling, respectively (reviewed in ref. 56). Due to the broad spectrum of signaling processes this receptor ties in, a reduced cell migration and cell-to ECM interaction seems to be a plausible consequence of the loss of CSPG4 expression. Since we observed co-expression of CSPG4 and CD271, a concerted action of both receptors may regulate melanoma cell properties. However, CSPG4 expression in metastases was not significantly higher as compared to primary melanoma. Although CD271 expression may facilitate melanoma cell migration, CD271 knock-down cells (BRAF wild type) retained a residual migratory capacity, suggesting that additional, CD271-independent factors determine melanoma cell migration. Since FGF13, CSPG4 and HMGA2 were not among the top up-regulated genes in the CD271^high^ subset of brain metastases, these factors may not be involved in melanoma brain metastasis.

The exploration of solitary and multiple brain metastases clearly demonstrated that CD271^+^ cells colonize and disseminate within the brain and are supposedly involved in distant and intracerebral metastasis. We observed that CD271^high^ BM showed a mutually exclusive expression of CD271 and MITF, hence a MITF^low^ phenotype. This was further supported by the finding that MITF-target genes were decreased in CD271^high^ tumors reasoning the low level of c-MET which is transcriptionally regulated by MITF^57^. Hence, CD271^high^ melanoma BM comprise a potentially individual subset with increased aggressiveness, increased DNA-repair capacity, stem-like properties or tendency to undergo EMT, and may respond differentially to therapeutic interventions. Moreover, the correlation of CD271 expression and PI3K/AKT pathway activation adds another distinct feature of CD271^high^ brain metastases.

In summary, our data suggest that CD271 expressing cells represent a population with brain metastatic capacity, capable of brain colonization and dissemination within the brain. Brain metastatic CD271^+^ cells feature increased DNA-repair, stem-like properties and low expression of MITF and MITF-targets, e.g. c-MET. Nevertheless, we observed CD271^+^ cells not exclusively in brain metastases but also in extracranial metastases of different sites. Hence, the question of how CD271^+^ cells prioritize the route of dissemination, remains. However, the high abundance of CD271^+^ cells in melanoma metastases suggests CD271 as a principal mediator of melanoma cell spreading. In fact, it has recently been shown in a mouse model system^10^ that targeting of CD271 may restrict or even prevent dissemination of primary melanoma. Besides CD271, other yet not fully understood factors like the interaction of melanoma cells with astrocytes or neuroinflammation^58^, seem to be prerequisites for the establishment of brain macrometastases.

## Material and Methods

### Patient cohorts

Archived (FFPE) matched pairs (n = 12) of extracranial (n = 13) and brain metastases (n = 12) including primary melanoma (n = 2) and unmatched extracranial (n = 1) and brain metastases (n = 7) were provided by the departments of pathology and neuropathology. Postmortem brains (n = 7) were taken 48 hours after death with informed consent from patients, and fixed in 4% neutral-buffered formalin for 2 weeks. Then brains were sectioned and tissue samples were taken for neuropathological examinations. Tumor samples were sectioned (2–4 µm) following paraffin embedding and stained for CD271. In addition hematoxylin-eosin (H&E) staining of sections was performed for histological expertise. All experimental methods were performed in accordance with the approved guidelines. The present study was approved by the ethics committee of the Charité - Universitätsmedizin Berlin (EA2/175/16).

### Cell culture

Patient-tumor derived cells (strain T20/02) as well as derivatives were cultured in Quantum 263 tumor growth medium (Q236, Capricorn Scientific, Germany) supplemented with 1% penicillin/streptomycin (Invitrogen, USA). A375 cells (ATCC CRL-1619, USA) as well as genetically engineered derivatives (GFP, CD271/NGFR) and MeWo cells (kind gift of Dr. Hermann Lage) were cultured in Leibovitz’s L-15 medium (Invitrogen) supplemented with 10% FBS, 1%, penicillin/streptomycin (Invitrogen), transferrin/fetuin, MEM-vitamines, insulin, glucose and sodium bicarbonate. Medium was changed every 3 days as described previously^27^.

### Organotypic slice cultures

Corticostriatal slice cultures were prepared from three-day-old mouse pups. Pups were sacrificed by instant decapitation. The brains were quickly removed and placed in a petri dish with ice cold Gibco Hanks’ Balanced Salt Solution. Both cerebral hemispheres were separated using a scalpel and sectioned coronally to 350 μm by a McIlwain tissue chopper (Mickle Laboratory, Cambridge, UK). The brain slices were separated under a dissection microscope (Leica S6 E) and placed on Millipore Millicell cell culture inserts (Millipore, Bedford, MA, USA) in a 6-well-plate with 1 ml culture medium (Gibco® RPMI + 10% heat inactivated FBS + 1% pen/strep). All experimental protocols were approved by the department of neuropathology of the Charité - Universitätsmedizin Berlin and all experimental methods were performed in accordance with the approved guidelines.

### Intracranial invasion assay

GFP/NGFR-GFP cells were washed with 5 ml of PBS. PBS was aspirated and 2 ml trypsin were added. The cells were incubated for 5 min, or until cells had detached, at 37 °C. 3 volumes of 10% FBS containing medium were added. The cells were centrifuged for 5 min at 1,100 rpm, medium was aspirated and cells were washed with PBS, a 50 μl aliquot of cells was saved for counting. After centrifugation, cells were re-suspended in fresh PBS to a concentration of 1 × 10^4^ cells/μl and transferred into a sterile Eppendorf tube. A WPI’s UltraMicroPump III (UMP3, World Precision Instruments, Sarasota, FL) and a 1 µl Hamilton microsyringe (Hamilton Company, Bonaduz, GR, Switzerland) were used to dispense 0.2 µl into the striatum. The injection was performed under a surgical microscope (Leica Wild M650) and GFP-positive tumor cells were visualized using fluorescent microscopy (Zeiss AxioObserver.Z1) every 24 hours. After 2 days the slices were fixed in 4% formaldehyde solution for 24 h at 4 °C.

### Immunohistochemistry

Formaldehyde-fixed and paraffin-embedded tumors were sectioned to 2 µm, following de-waxing, peroxidase blocking and antigen-retrieval using EDTA buffer. Detection of CD271, CD31, Ki67, S100, vimentin, MITF, HMB45 and MART1/MLANA was performed with anti-CD271 (clone D4B3, XP, Cell signaling), anti-CD31 (clone JC/70A, Dako), anti-Ki67 (clone MIB1, Dako), anti-S100 (clone 15E2/E2, BioGenex), anti-vimentin (clone V9, Dako), anti-MITF (clones C5 + D5, Zytomed), anti-melanosom (clone HMB45, Dako) or anti-MART1 (clone A103, Dako) with an automated staining system (BenchMark Ultra, Ventana Medical Systems, USA). Histological staining was performed with hematoxylin/eosin (H&E, Merck). Immunohistochemical staining of two antigens was performed with the DakoCytomation kit (Dako).

### Immunofluorescence and Confocal microscopy

Paraformaldehyde (PFA, 4%) fixed brain slices were mechanically removed, blocked (2% bovine serum albumin, BSA) and stained with either fluorescently (Phycoerythrin, PE) labeled or unlabeled anti-CD271 (Miltenyi, clone ME20.4–1.H4, mouse IgG1, or clone D4B3, XP, rabbit, 1:100, Cell signaling) and DAPI (4′,6-Diamidin-2-phenylindol, Sigma-Aldrich, 1:500), for 24 hours at 4 °C. Images were captured using Zeiss Axiovert40CFL with accompanied Illuminator HPX120C and software AxioVision Rel. 4.8 (all Carl Zeiss AG, Germany). Confocal images were captured with Leica TCS SP5 (Leica, Germany) using sequential scanning (z-stacking) at 200 Hz scan speed and 0.5 µm or 1 µm/stack. For immunofluorescence of tumors, sections were de-waxed followed by antigen-retrieval (citrate buffer pH = 6.0) for 20 min, washed in tris-buffered saline (TBS) and blocked in phosphate-buffered saline (PBS) with 2% bovine serum albumin (BSA) for 1h. Sections were incubated with blocking buffer and primary antibodies vimentin (mAB, clone V9, Invitrogen, Carlsbad, USA) and CD271 (clone D4B3, XP, Cell signaling) for 1 h at RT or overnight at 4 °C. Sections were incubated for 1 h at RT with secondary antibodies AlexaFluor488 and 594 on second day, following washing of with PBS/Tween20 (0.1%). For cell-based immunofluorescence, cells were fixed directly on well plates, blocked with PBS/BSA and incubated with directly labeled antibodies CSPG4-FITC or CD271-PE (Miltenyi, Germany) or phalloidin-TRITC (Invitrogen) for 1h at RT.

### BrdU labeling

Following incubation, cells were fixed with PFA (4%/PBS), permeabilized using triton-X100 (0.1%/PBS) for 10 minutes and incubated with hydrochloric acid (2.5 M) for 30 minutes at RT. Following, cells were blocked with BSA and stained with anti-BrdU or Ki67 antibody (0.5 µg/ml final, BD Bioscience, Cat. No. 347580 or abcam, Cat. No. 66155) for 1 h at room temperature (RT). Secondary antibodies (anti-mouse AlexaFluor594 (BrdU) or 488 (Ki67), final concentration 2ug/ml) were added and incubated for 1 hour at RT.

### Immunoblot

Total cell lysates were prepared in SDS lysis buffer (1% SDS, 10 mM Tris-HCl, 2 mM EDTA). 10–25 µg of whole cell lysates were separated on 12% SDS-PAGE gels and transferred on a nitrocellulose membrane by using the iBlot Dry Blotting System (Invitrogen). Membranes were blocked with Odyssey Blocking Buffer/PBS-Tween (0.1%), (1:1, LI-COR Biosciences, USA) for 1 h. Incubation with primary antibodies (p75NTR clone D4B3XP® recognizing the total CD271, p-ERK1/2, ERK1/2, p-AKT(S473), AKT, and β-tubulin, clone 9F3; all 1:1000; from Cell Signaling Technology, Germany) was done overnight at 4 °C. Incubation with secondary antibodies donkey anti-rabbit IgG or donkey anti-mouse both (diluted 1:10000; LI-COR Biosciences, USA) was done for 1 h at RT. Signals were detected with the Odyssey Infrared Imaging System (LI-COR Biosciences, USA).

### RNA isolation and qRT-PCR

RNA isolation of frozen cell pellets was performed with the RNeasy Mini Kit (Qiagen, Germany), following the manufacturers protocol. Reverse transcription of 2.0 µg total RNA was performed with the SuperScript VILO cDNA synthesis kit (Invitrogen, Germany) and diluted to a final volume of 50 µl. qRT-PCR was carried out on a Step one plus (Applied Biosystems, Germany) for 40 cycles. Primers were designed using the PrimerQuest Tool (http://eu.idtdna.com/ primerquest/home/index) for 55–60 °C annealing temperature and product size of 100–250 bp (SI, Table S4). qPCR expression levels were calculated as relative expression by ΔΔCT, normalized to HPRT (hypoxanthine phosphoribosyltransferase 1).

### Gene-set enrichment analysis (GSEA)

GSEA was performed with appropriate software (GSEA version 2.2.2) as well as signatures provided by the Broad institute (http://software.broadinstitute.org/gsea/index.jsp). Signatures specific for cell projections, focal adhesion, melanoma aggressiveness, DNA-repair, neural crest stem cells (NCSC) and EMT were received from the latest version of the Molecular Signatures Database v5.1 (MSigDB) of the Broad institute. Derivation of the CD271-responsive gene signature was performed as described previously^27^. Genes for signatures specifying MITF-targets and brain metastatic cells were derived from publications^14, 43^. Expression data of primary melanoma, extracranial metastases and brain metastases were obtained from GEO (Gene expression omnibus).

### Data availability

The datasets analysed in the current study are available in the GEO database with accession numbers GSE7553 (skin cancer subtypes), GSE46517 (melanoma progression), GSE50493 (melanoma METs including brain METs, set 1), GSE44660 (brain METs set 2), GSE15605, GSE8401 (PM, MET), GSE52456 (shCD271, melanoma), GSE43741 (shHMGA2, breast cancer). CD271-responsive genes were extracted from data sets GSE78155: T20/02 (CD271/NGFR; Mock) A375 (CD271/NGFR; GFP) as well as GSE52456 (shCD271, T20/02) as reported previously^32^.

### Meta-analysis

Pre-processed data (normalized, batch-corrected) were imported to excel, log2 transformed and filtered for significance p ≤ 0.05, determined by a two-sided, two-sample ttest for unequal variance. Tables containing significant expression data (log2) were imported in R-studio (R Ver. 3.3.2) and tested for Gaussian distribution (p ≥ 0.05) by Shapiro testing. Tumor samples mostly did not show a Gaussian distribution and p-values were determined by a Wilcoxon rank-sum test. Box plots were generated using the box plot function of the ggplot2 package in combination with the beeswarm function. Heat maps were generated with heat map function of the gplot package. Brain and extracranial metastases with highest or lowest expression were designated as CD271^high^ or CD271^low^, respectively. For identification of commonly regulated genes, lists containing either most up- or down-regulated genes were compared with R’s merge function. Duplicate gene symbols were removed by data filtering in excel. Due to the low sample number of brain metastases (n = 7) of study GSE44660 p-values were determined by ttest.

## Electronic supplementary material

Supplementary information (SI)

Movie S1

Movie S2

Movie S3

Movie S4

Movie S5

Movie S6

Movie S7

Movie S8
