# Supplementary material for: CD271 determines migratory properties of melanoma cells
Source: Sci Rep. 2017 Aug 29;7:9834. doi: 10.1038/s41598-017-10129-z (PMC5574914; doi:10.1038/s41598-017-10129-z)
Supplement: Supplementary file 1 — Supplementary information (SI) [file 41598_2017_10129_MOESM1_ESM.doc]

**CD271 determines migratory properties of melanoma cells**

Josefine Radke, Florian Rossner and Torben Redmer

**Supplementary information**

**Figures**

**Figure S1: Pattern of CD271 expression in melanoma metastases (1).** Depiction of melanoma patients analyzed for expression and phenotypic appearance of CD271 positive cells in matched primary tumors (PM) or extracranial metastases (EM) and brain metastases (BM).

**Figure S2: Pattern of CD271 expression in melanoma metastases (2). (A)** Depiction of melanoma patients analyzed for expression and phenotypic appearance of CD271 positive cells in matched extracranial (EM) and brain metastases (BM). **(B)** Depiction of unmatched brain metastases for expression and phenotypic appearance of CD271 positive cells.

**Figure S3: Heterogeneity of desmoplastic neurotrophic melanoma (DNM). (A)** Upper row: Comparison of CD271 expression of primary tumor (Primary) as well as two skin metastases (A, B; Patient 1) or hematoxylin/eosin (H&E), lower row. **(B)** Vimentin expression is typical for desmoplastic neurotrophic melanoma. Two areas (1,2) depict the heterogeneity of the same tumor (upper panels). Immunofluorescence microscopy of CD271 (green) and vimentin (red) depicts the cellular heterogeneity of the same tumor area (lower panels). Scale bars indicate 50 µm. DAPI served as nuclear stain.

**Figure S4: Expression of CD271 in healthy and tumor adjacent tissue. (A)** Upper and center rows: Expression of CD271 in cells of the basement membrane in healthy skin as well as in MART1 positive melanocytes, showing either a proliferative, Ki67^+^ or non-proliferating (Ki67^-^) phenotype. Lower row: Co-immunohistochemistry of CD271 (red) and Ki67 or MITF (both brown). **(B)** Expression of CD271 in adjacent tissue of a lymph node (LN), infiltrated by tumor cells (T).

**Figure S5: Comparative analysis of melanoma liver and brain metastases indicates occurrence of CD271 at different metastatic sites. (A)** Immunohistochemistry of a uveal melanoma demonstrates a high expression of CD271 in the tumor adjacent choroid as well as in spindle-like (T, Spin) but not epitheloid tumor (T, Epi) cells (Patient 10). **(B)** Upper row: Phenotypical appearance and CD271 expression of tumor cells of a liver metastasis of the uveal melanoma described in (A). CD271^+^ cells were observed in tumor (T) and adjacent tissue (liver parenchyma, LP). Center and lower rows: Exploration of the liver metastasis described in (B) for presence of CD271 and the hepatocyte marker Hep.par1. (**C**) Exploration of a brain metastasis (BM) of the uveal melanoma (A) for expression of CD271 and H&E.

**Figure S6: Melanoma subtypes show distinct levels of CD271 expression. (A)** Exploration of melanoma subtypes (GSE15605) for expression levels (log2) of CD271/NGFR demonstrates a subtype-dependency of expression starting from normal skin (NS) and melanoma in-situ (MIS) to aggressive melanoma subtypes: acral (ACRAL), superficial spreading melanoma (SSM), nodular melanoma (NODULAR), desmoplastic melanoma (DESMOPLASTIC), lentigo maligna melanoma (LMM) and spindle cell melanoma (SPINDLE CELL); regional lymph node metastases (REG. LN MET), distant metastases (DISTANT MET) and subcutaneous soft tissue metastases (SC_ST MET). **(B)** Expression levels (log2) of CD271 (NGFR) in brain metastases of studies GSE50493 and GSE44660 subclassified into CD271^high^ (n=5; n=3) and CD271^low^ (n=13; n=4) tumors. **(C)** Supervised clustering of CD271^high^ (red) and CD271^low^ (green) tumors of 416 differentially regulated genes among them 160 up-regulated genes (FC_log2_≥1.5, p≤0.05) and 256 down-regulated genes (FC_log2_≤1.5, p≤0.05). **(D)** Venn-diagram depicts genes commonly regulated among CD271^high^ and CD271^low^ brain tumors of studies mentioned in (B). Duplicate gene symbols were removed. **(E)** Individual commonly regulated genes identified in (D) were validated for expression levels in CD271^high^ (blue) and CD271^low^ (red) tumors. In (B) and (E), p-values shown were determined by Wilcoxon rank-sum test.

**Figure S7: CD271^high^ brain metastases show expression of CD271-responsible and stem-like genes. (A)** Gene-set enrichment analysis (GSEA) of CD271^high^ (BM-CD271^high^) vs. CD271^low^ (BM-CD271^low^) brain metastases of study GSE50493 revealed enrichment of genes associated with DNA-repair (left panel), a neural crest stem-like state (NCSC, center panel) or formation of cell projections (right panel) in the CD271^high^ subgroup. **(B)** Left panel: GSEA of samples in (A) revealed enrichment of genes found in brain metastatic and vemurafenib-resistant melanoma cells. Right panels: GSEA of samples described in (A) but of study GSE44660 revealed enrichment of genes associated with epithelial-to-mesenchymal transition (EMT) and CD271-responsible genes. **(C)** Over expression of CD271 induces formation of lamellopodia. A375^GFP^ and A375^CD271/NGFR^ cells were stained for CD271 (red, left column) or phalloidin (red, center and right columns), showing proper expression of CD271 in A375^CD271/NGFR^ cells and a rear-to-front polarity induced by over expression of CD271.

**Figure S8: A375^GFP^ and A375^CD271/NGFR^ cells show intracranial proliferation in mouse brain slices. (A)** Imaging of A375 cells stably expressing GFP or CD271/NGFR (GFP), 24, 48, 96 and 110h post intracerebral injection into 350 µm mouse brain slices. **(B)** Exploration of CD271^+^ cells revealed the presence of cells migrating from the potential invasive front through the brain metastasis (first columns) or away into adjacent brain parenchyma (last column) in a selected patient tumor sample.

**Figure S9:** **A375^GFP^ cells show intracranial migration in murine organotypic brain slices. (A)** Serial confocal imaging of mouse brain slices 48h following injection of A375^GFP^ cells. Images depict the sequential migration of cells away from the area of injection, passed through the slice. Lower row: Distinct or co-staining of long-distance migration cells accumulated in the last section (Z14/14) for CD271 and GFP. **(B)** Same imaging as performed in (A) but of an independent injection experiment. White dotted lines indicate the initial border of the tumor mass, DAPI served as nuclear stain, scale bars indicate 50 µm. Yellow dotted lines in Z0, Z7 and Z14 indicate the distance of migrated cells. Around 0.2 - 0.4 % of A375^GFP^ cells showed long distance migration (534 µm).

**Figure S10:** **A375^CD271/NGFR^ cells show intracranial migration in murine organotypic brain slices. (A)** Confocal imaging of mouse brain slices 48h following injection of A375^CD271/NGFR^ cells. Images depict the sequential migration of cells away from the area of injection, passed through the slice. Lower row: Distinct or co-staining of long-distance migration cells accumulated in the last section (Z7/7) for CD271 and GFP. **(B)** Same imaging as performed in (A) but of an independent injection experiment. Magnified areas of cells passed through the slice after 48h show a lamellopodia-like structure and staining of CD271. White dotted lines indicate the initial border of the tumor mass, DAPI served as nuclear stain, scale bars indicate 50 µm.

**Figure S11: Knock-down of CD271 induces S-phase arrest in melanoma cells.** **(A)** Left panels: BrdU incorporation of control cells (shCtl.) or knock-down cells (shCD271), following incubation with bromo-5´-deoxyuridine (BrdU, 20 µM final) for 30 minutes. **(B)** Quantification of BrdU^+^ cells relative to DAPI. P-value was determined by a two-sided, two-sample ttest for unequal variance.

**Figure S12: Migration of CD271^+^ cells in the murine organotypic brain slices. (A)** Representative confocal microscopy images of brain slices 5d post injection of knock-down control (shCtl., T20/02) cells followed by staining with CD271. Shown are CD271 (red) and DAPI in z-stack images Z0 and Z7 of 1.0 µm stacks (first columns) as well as magnified areas depicting radial and vertical migration. **(B)** Imaging of CD271 knock-down cells (sh#4) stably expressing GFP in brain slice cultures, 24, 48, 72 and 96h post intracerebral injection. Slices were harvested after 5 days and processed as previously described. **(C)** Representative confocal microscopy images from z-stacking as described in (B) showing residual migratory activity.

**Figure S13: CD271 acts in concert with HMGA2 to regulate migration associated genes and is associated with active AKT-signaling. (A)** Exploration of HMGA2 expression of primary melanoma (PM, brown/grey) and metastases (MET, red/green) of two independent studies (GSE46517, GSE7553). **(B)** Identification of HMGA2/CD271-responsible genes by comparative analysis of genes down-regulated either by knock-down of HMGA2 in a breast-cancer cell line (MDA-MB-231, GSE43741) or by knock-down of CD271 in a melanoma cell line (T20/02, GSE52456). Among the identified, commonly regulated genes (n=130, FC_log2_≤0.5, p≤0.05) were mediators of migration. **(C)** Exploration of expression levels of individual indentified migration-associated HMGA2/CD271-responsible genes in primary melanoma and metastases; PDZ And LIM Domain 1 (PDLIM1), semaphorin 3A (SEMA3A), ST3 beta-galactoside alpha-2,3-sialyltransferase 1 (ST3GAL1) and lysyl oxidase (LOX). **(D)** Left and center panel: Exploration of levels of CSPG4 in primary tumors and metastases of studies mentioned in (A). Right panels: immunofluorescence microscopy of melanoma cells (MeWo, parental; MeWo^Par^ and T20/02) for distinct and co-localization of CD271 (red) and CSPG4 (green). DAPI served as nuclear stain, scale bars indicate 50 µm. **(E)** Exploration of levels of FGF13 in primary tumors and metastases of studies mentioned in (A). In (A), (C), (D) and (E), p-values were determined by Wilcoxon rank-sum test. **(F)** Left panels: Immunoblot analysis of whole cell lysates of melanoma cells (T20/02) stably transfected with CD271-targeting shRNAs (sh#3, sh#4; sh#2: not effective) or control shRNA (shCtl.) or untransfected (Mock) for levels of phosphorylation-activated AKT (pAKT^S473^), total AKT (AKT), phosphorylation-activated ERK1/2 (pERK1/2), total ERK (ERK) as well as CD271. Right panels: Immunoblot analysis of whole cell lysates of melanoma cells mentioned before but FACS-sorted for high (+) or low (-) expression of CD271, or non-sorted (Bulk) for proteins analyzed before. β-tubulin or ERK1/2; AKT served as loading controls.

**Figure S14: SEMA3A and FGF13 respond to levels of CD271 expression. (A-B)** Expression of SEMA3A, FGF13, HMGA2 and PDLIM1 was analyzed in melanoma metastases (MET, blue) subclassified into CD271^high^ or CD271^low^ and primary tumors (PM, red). Expression of SEMA3A and FGF13 (Responding) but not HMGA2 and PDLIM1 (Non-responding) responded to CD271 levels. P-values were determined by Wilcoxon rank-sum test. **(C)** Schematic representation of the potential regulation of migration-associated genes by CD271 alone or via HMGA2 or FGF13.

**Figure S15: CD271 expressing cells reside in multiple brain metastases of melanoma patients. (A) Upper rows:** Immunohistochemistry of sections of autopsied multiple brain metastases of patient T1 shows high abundance of CD271^+^ cells in a metastasis of the frontal cortex. Shown is the second metastasis, located in the frontal left cortex. In the healthy brain parenchyma, neurons show CD271 positive staining. Lower rows: Exploration of brain metastases of patient T1 located in the frontal cortex or cerebellum for melanoma markers MART1/MLANA, HMB45 and S100 (all Agilent technologies, CA, USA). **(B)** Upper rows: Sections of brain metastases located in the temporomesial cortex and left cerebellum (Patient T2) stained for CD271 and melanoma markers (lower rows).

**Figure S16: CD271^+^ cells reside in micrometastases and are found adjacent to blood vessels. (A)** Identification of CD271^+^ cells in micrometastases in the cerebellum and hippocampus (Patient T3). **(B)** CD271^+^ melanoma cells are located in close proximity to blood-vessels as shown by co-immunohistochemistry of CD271 and CD31 in a brain metastasis (Patient 1). Two areas (1,2) are shown. **(C)** CD271^+^ cells in a blood vessel within a brain metastasis of (A). **(D)** Potential migration of CD271^+^ brain metastasis cells (Patient T1) to blood vessels. Distinct (CD271, CD31; brown) and double staining (CD271, red/ CD31, brown) is shown.

**Tables**

**Table S1: Intra-tumor heterogeneity of CD271 expression.** Summary of tumor specimens analyzed for expression of CD271. Shown is additional information of primary tumors (n=2) matched pairs of extracranial (EM, n=13) and brain metastases (BM, n=12 of melanoma patients as well as unmatched extracranial (n=1) and brain metastases (n=7). The percentage of CD271 and MART1 positive cells, localization of CD271 expression, melanoma subtype (MM), BRAF status, sites of metastasis at diagnosis and, if identified, site of brain tumor metastases as well as additional sites of metastases under therapy is shown. The present study was approved by the ethics committee of the Charité - Universitätsmedizin Berlin (EA2/175/16).

**Table S2: Concordance of CD271 expression in multiple brain metastases.** The table summarizes the regions of autopsied multiple brain metastases, location of additional extracranial metastases and provides information about the last therapy regimen as well as the CD271 expression.

**Table S3: Potential HMGA2/CD271-responsible genes.** Potential targets of HMGA2/CD271 were identified by a comparative analysis of genes down-regulated (FC_log2_≤0.5, p≤0.05) either by shHMGA2 in MDA-MB-231 (breast cancer) or shCD271 in T20/02 (melanoma) cells.

**Table S4: qPCR primers.** Shown are sequences of qPCR primers, designed for SybrGreen-based PCR as described previously[^1^](#_ENREF_1).

**Movies files**

**Movie S1**: A375^GFP^: migration of GFP^+^ cells (green), 48 h after injection in mouse brain slices and sequential confocal microscopy.

**Movie S2**: A375^GFP^: migration of CD271^+^ cells (red), 48 h after injection in mouse brain slices and sequential confocal microscopy.

**Movie S3**: A375^CD271/NGFR^: migration of CD271^+^ cells (red), 48 h after injection in mouse brain slices and sequential confocal microscopy.

**Movie S4**: Live cell-imaging of residually migrating CD271 knock-down cells (T20/02, shCD271) is shown. Imaging of scratch wounds (n=8, a representative is shown) was performed every 3 hours.

**Movie S5**: Live cell-imaging of migrating knock-down control cells (T20/02, shCtl.) is shown. Imaging of scratch wounds (n=8, a representative is shown) was performed every 3 hours.

**Movie S6**: Live cell-imaging of migrating GFP-expressing T20/02 cells. Imaging of scratch wounds (n=8, a representative is shown) was performed every 3 hours.

**Movie S7**: Migration of CD271^+^ shCtl. cells (T20/02, red), 5d after injection in mouse brain slices and sequential confocal microscopy.

**Movie S8**: Partially migration of GFP^+^/CD271^-^ shCD271 (sh#4) cells (T20/02, green), 5d after injection in mouse brain slices and sequential confocal microscopy.

**References**

1 Redmer, T. *et al.* The nerve growth factor receptor CD271 is crucial to maintain tumorigenicity and stem-like properties of melanoma cells. *PloS one* **9**, e92596, doi:10.1371/journal.pone.0092596 (2014).

|  |
| --- |

| 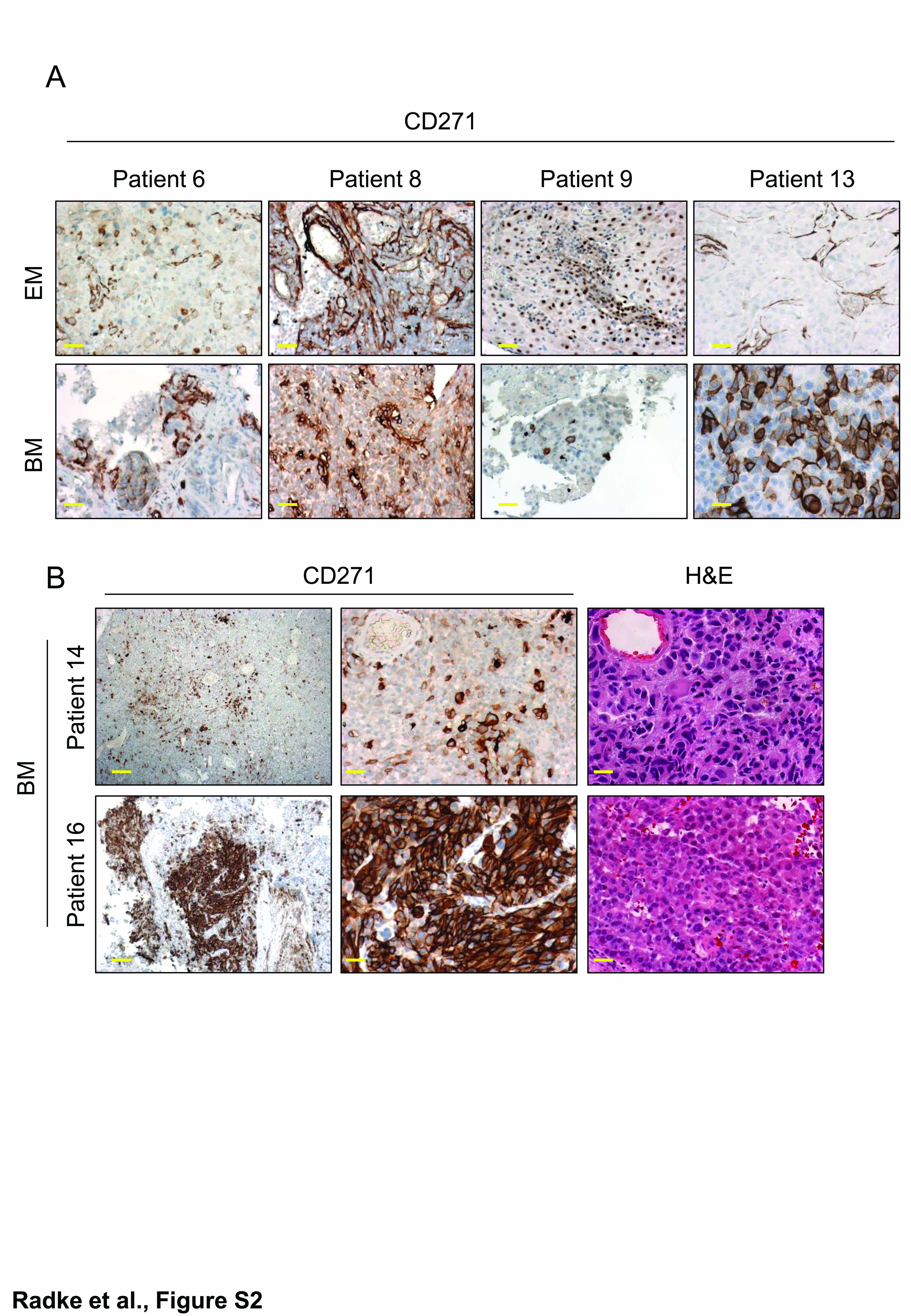 |
| --- |

| 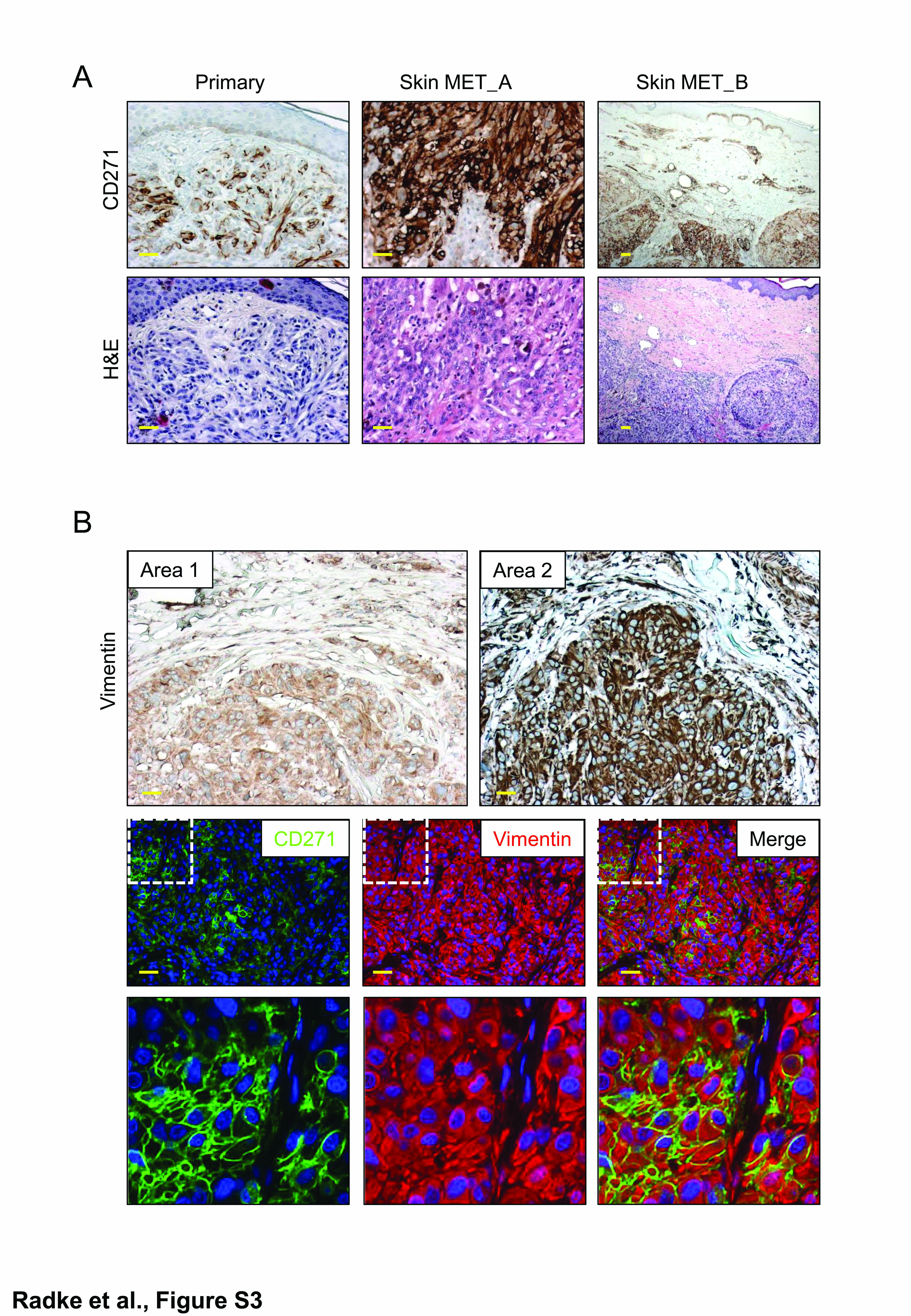 |
| --- |

| 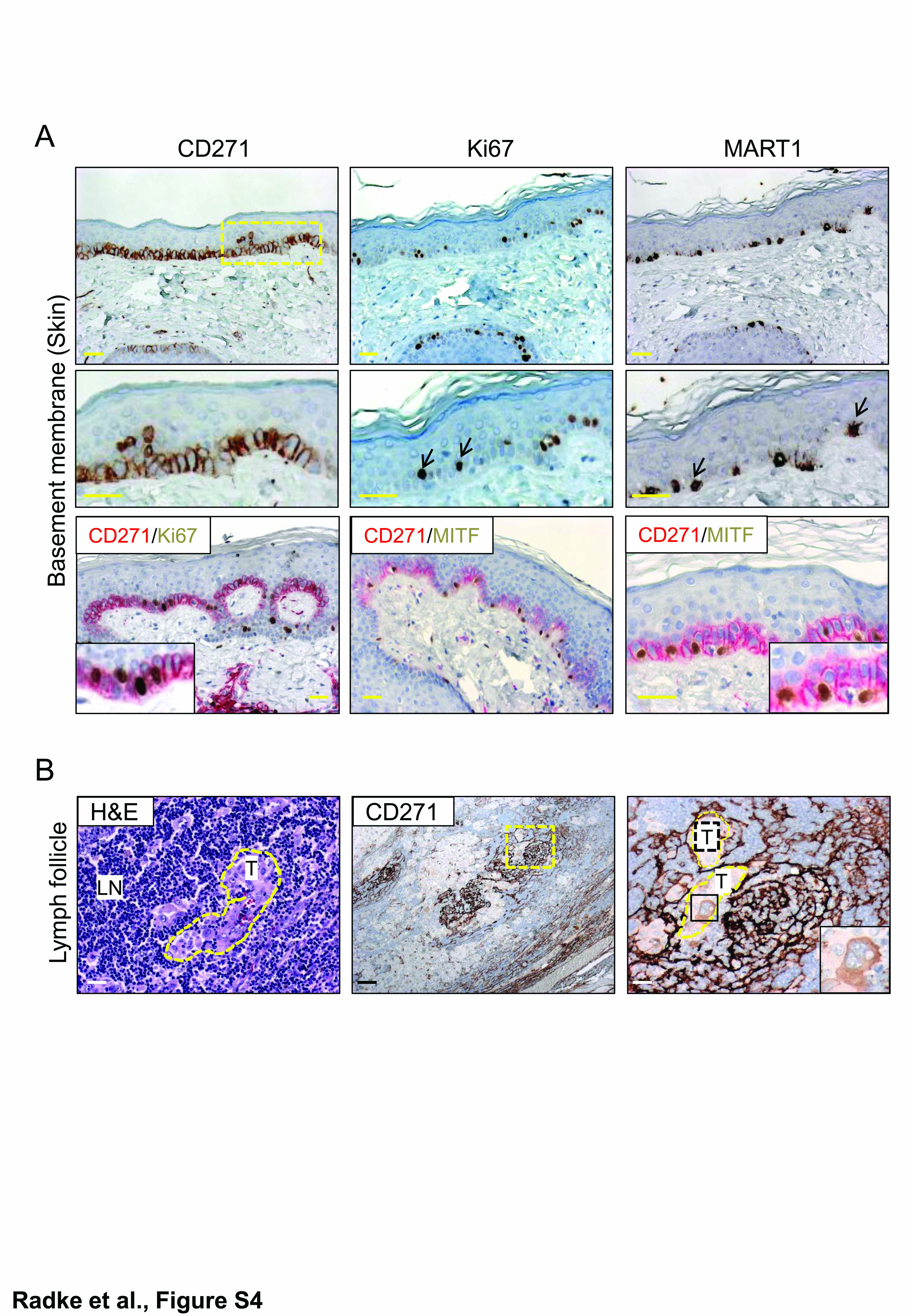 |
| --- |

| 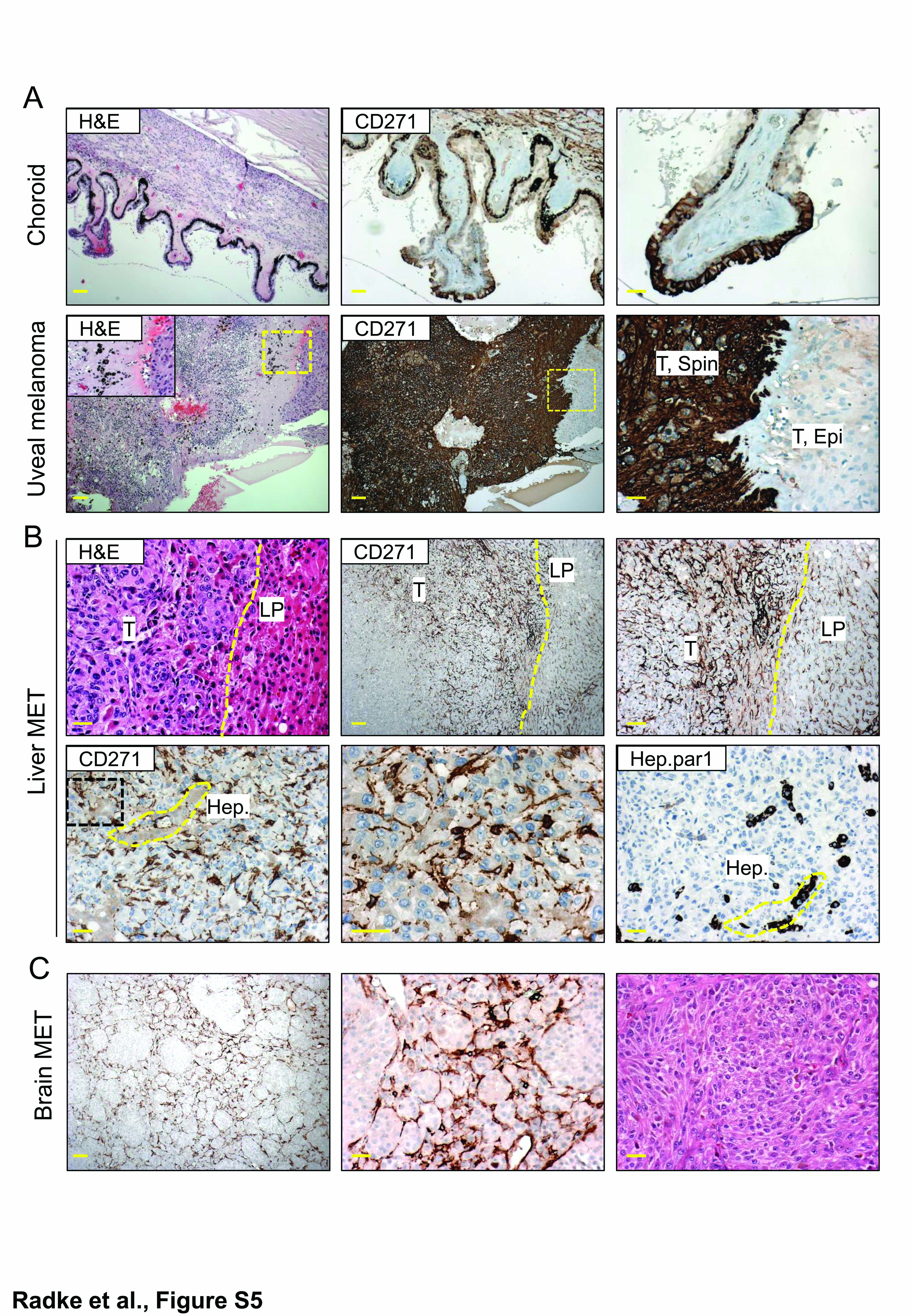 |
| --- |

| 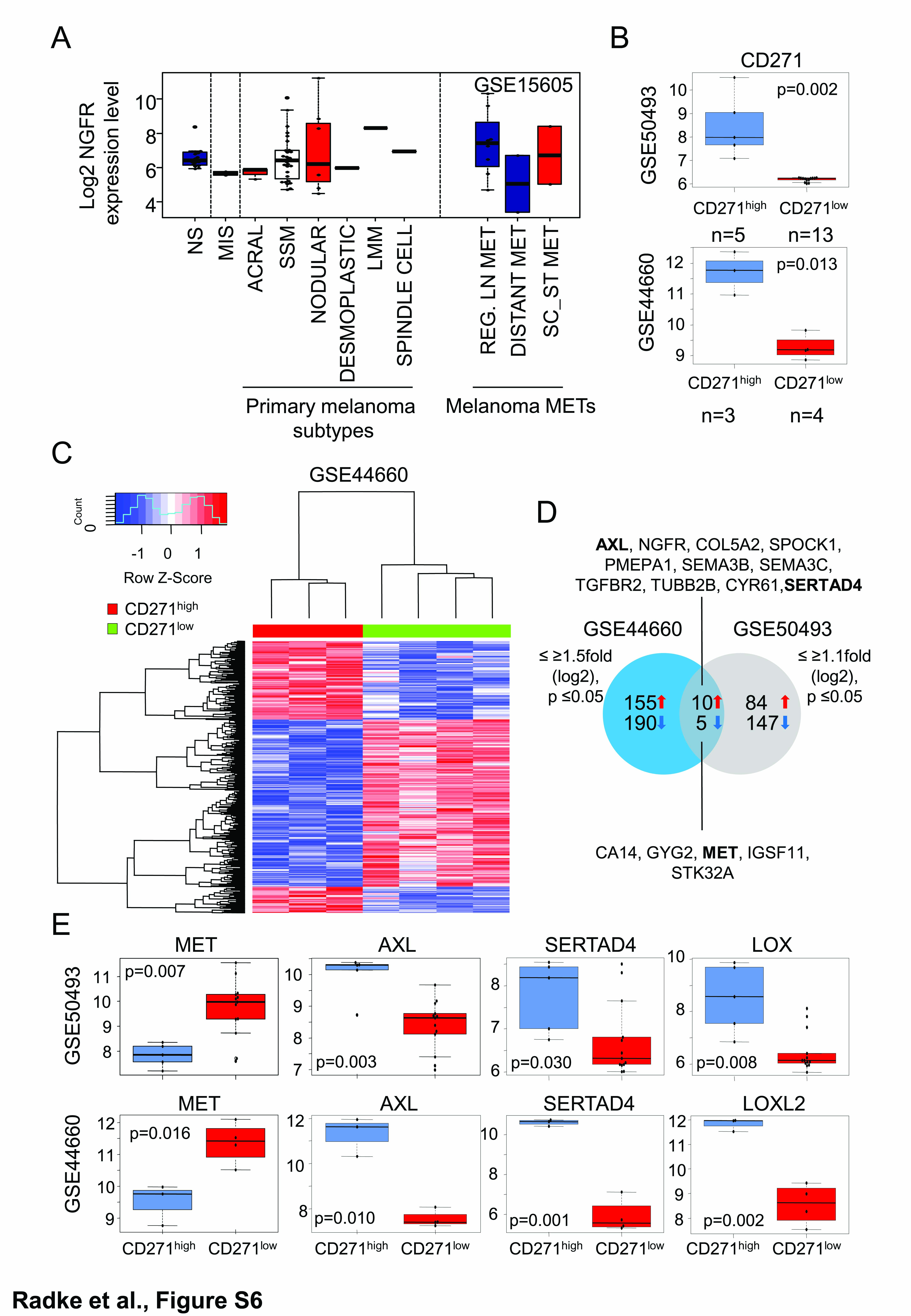 |
| --- |

| 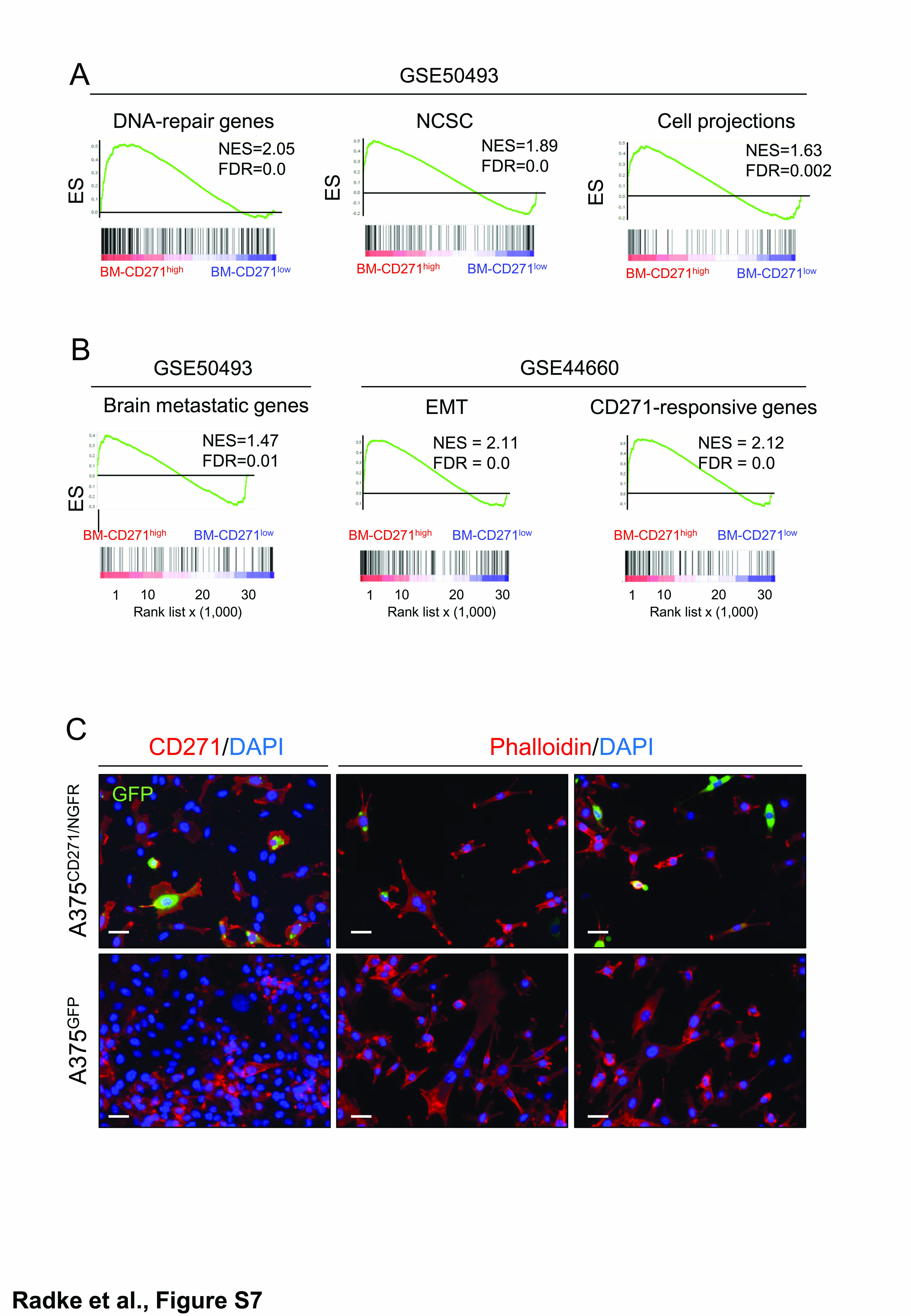 |
| --- |

| 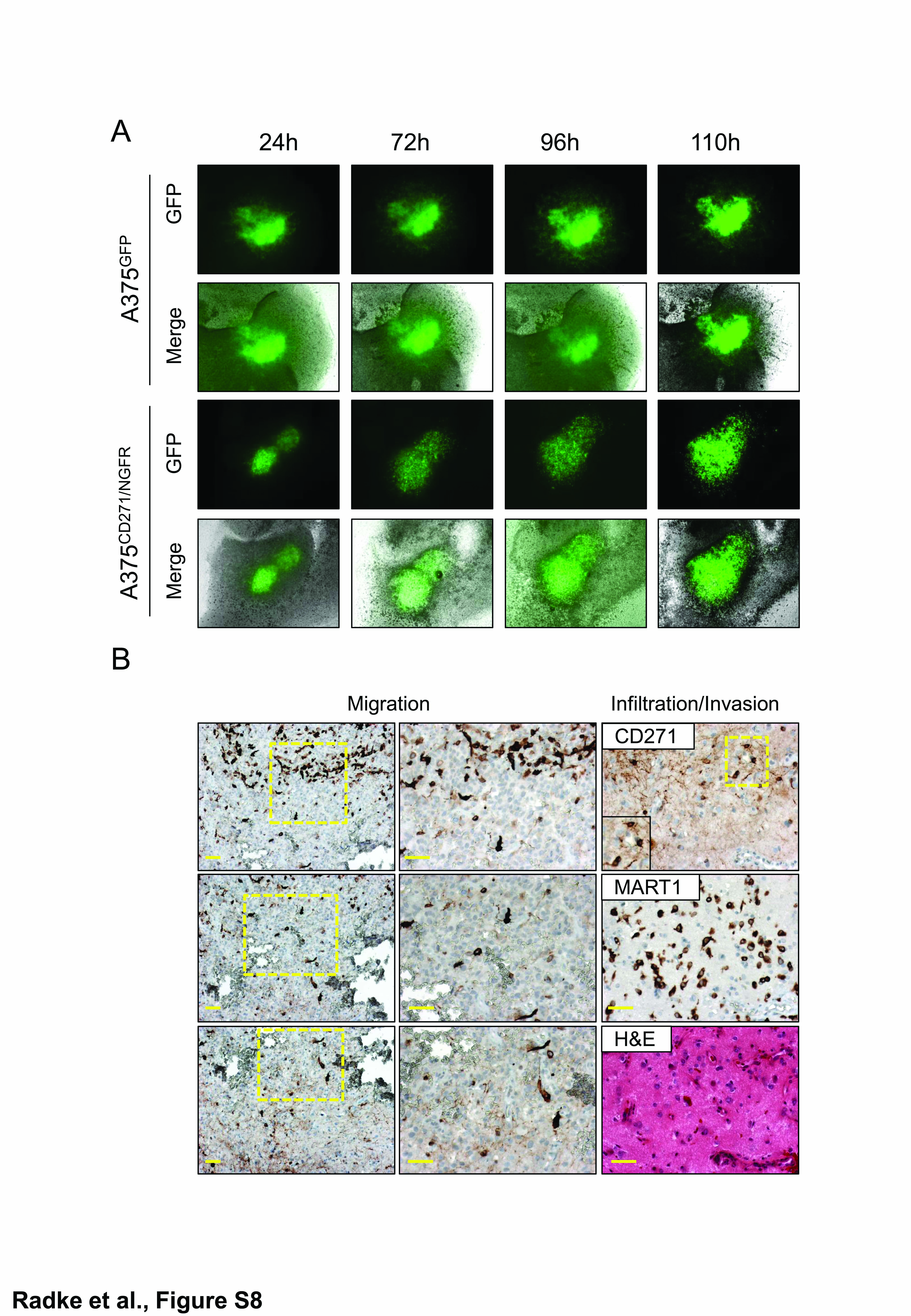 |
| --- |


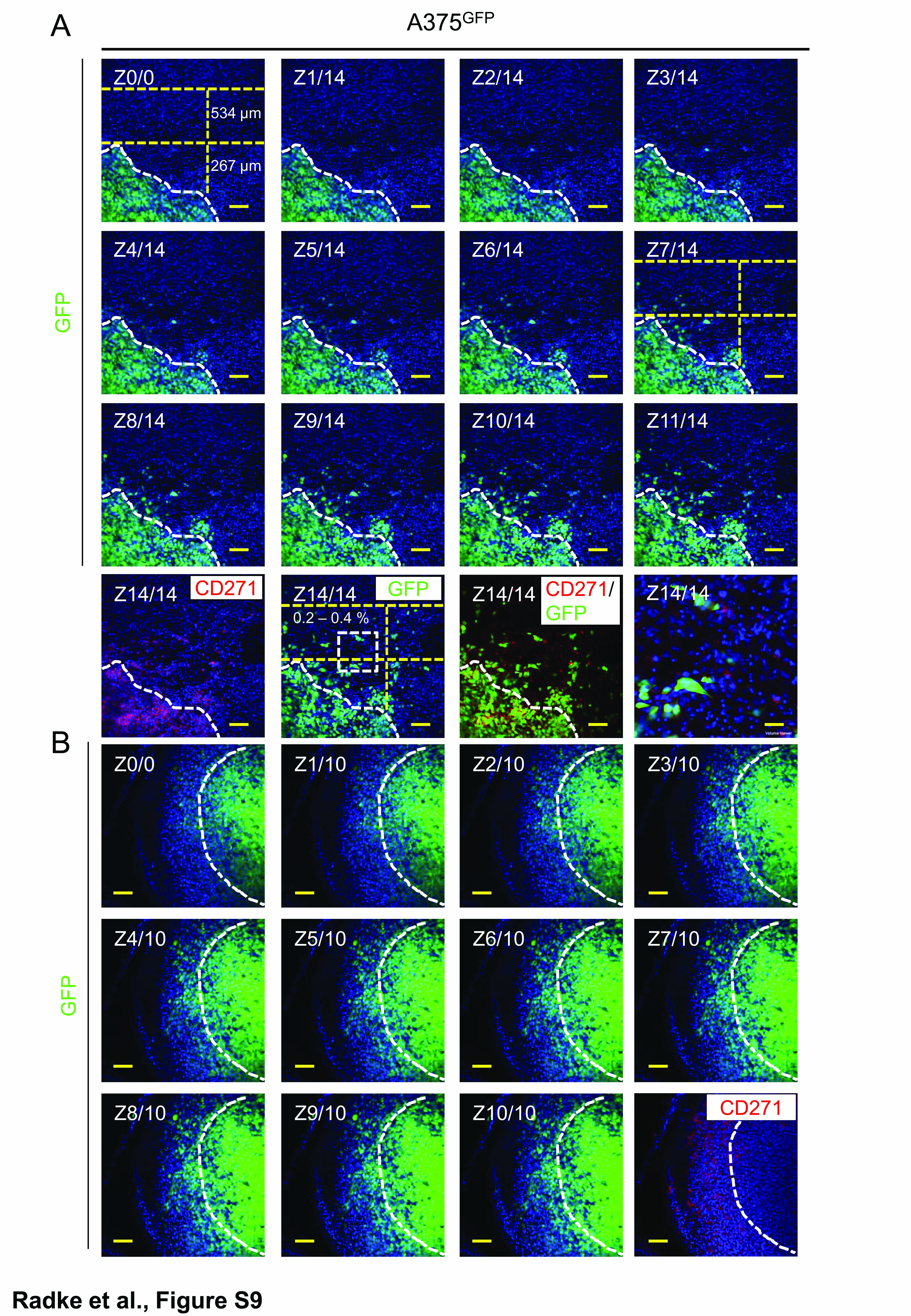


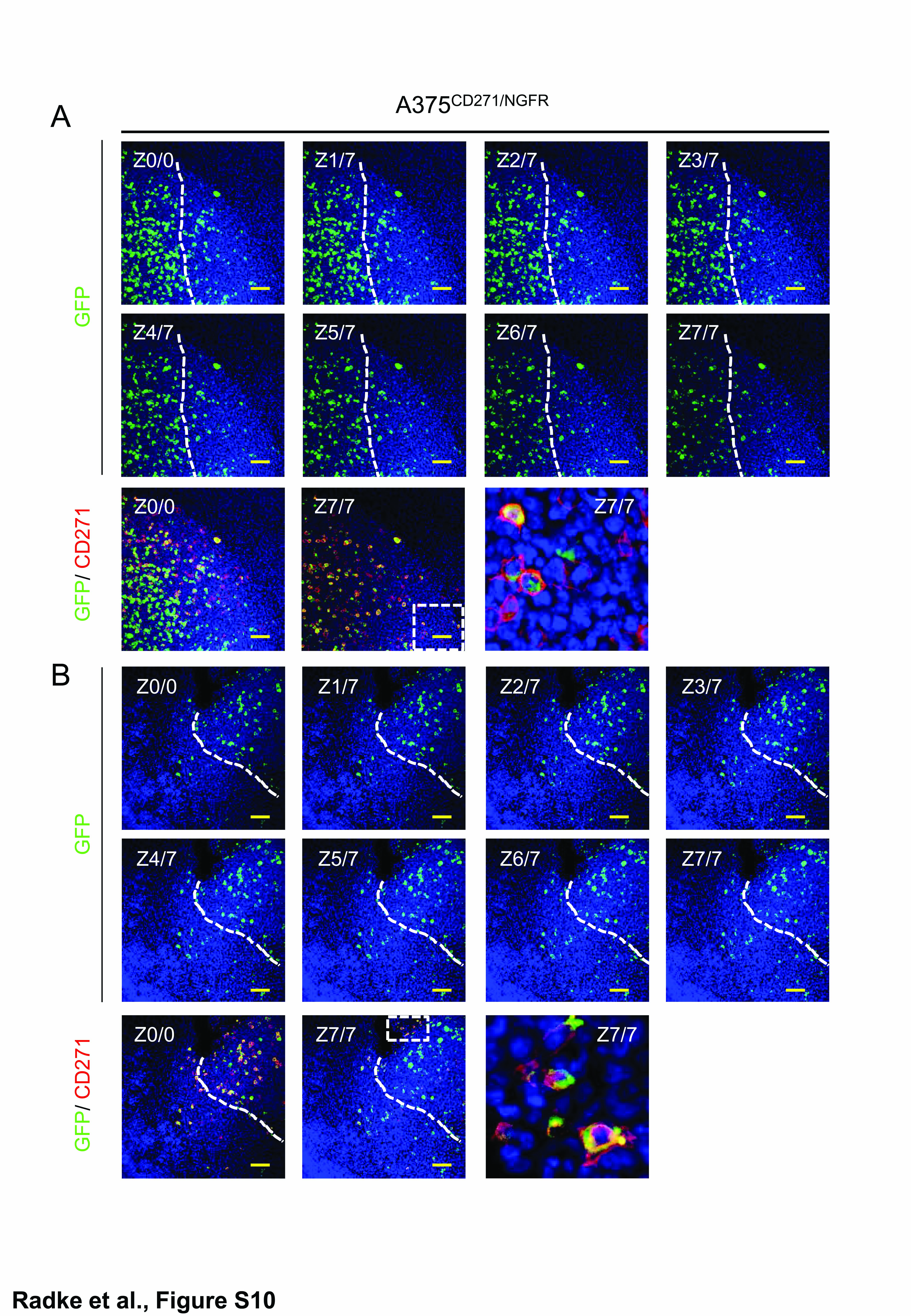


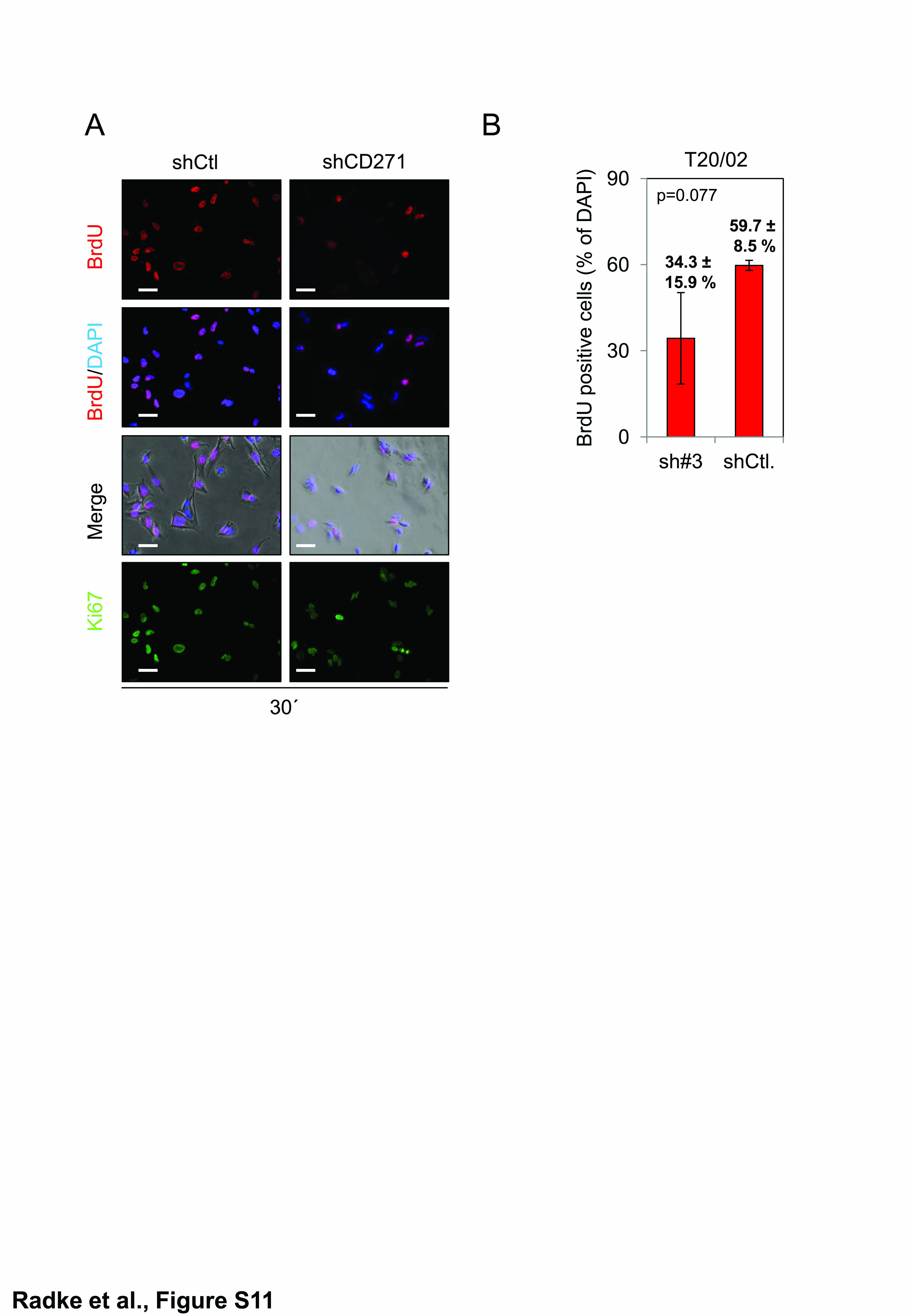


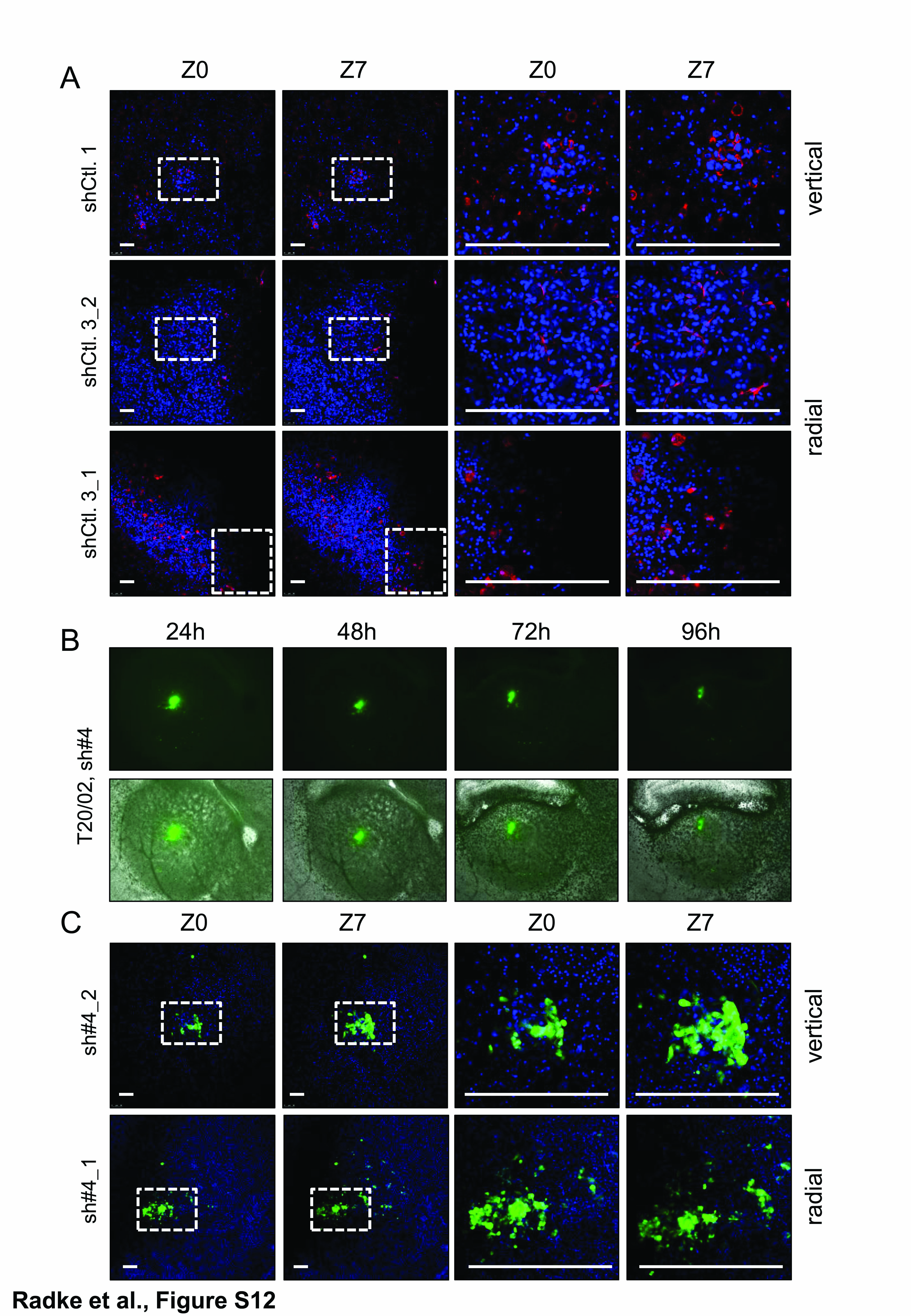


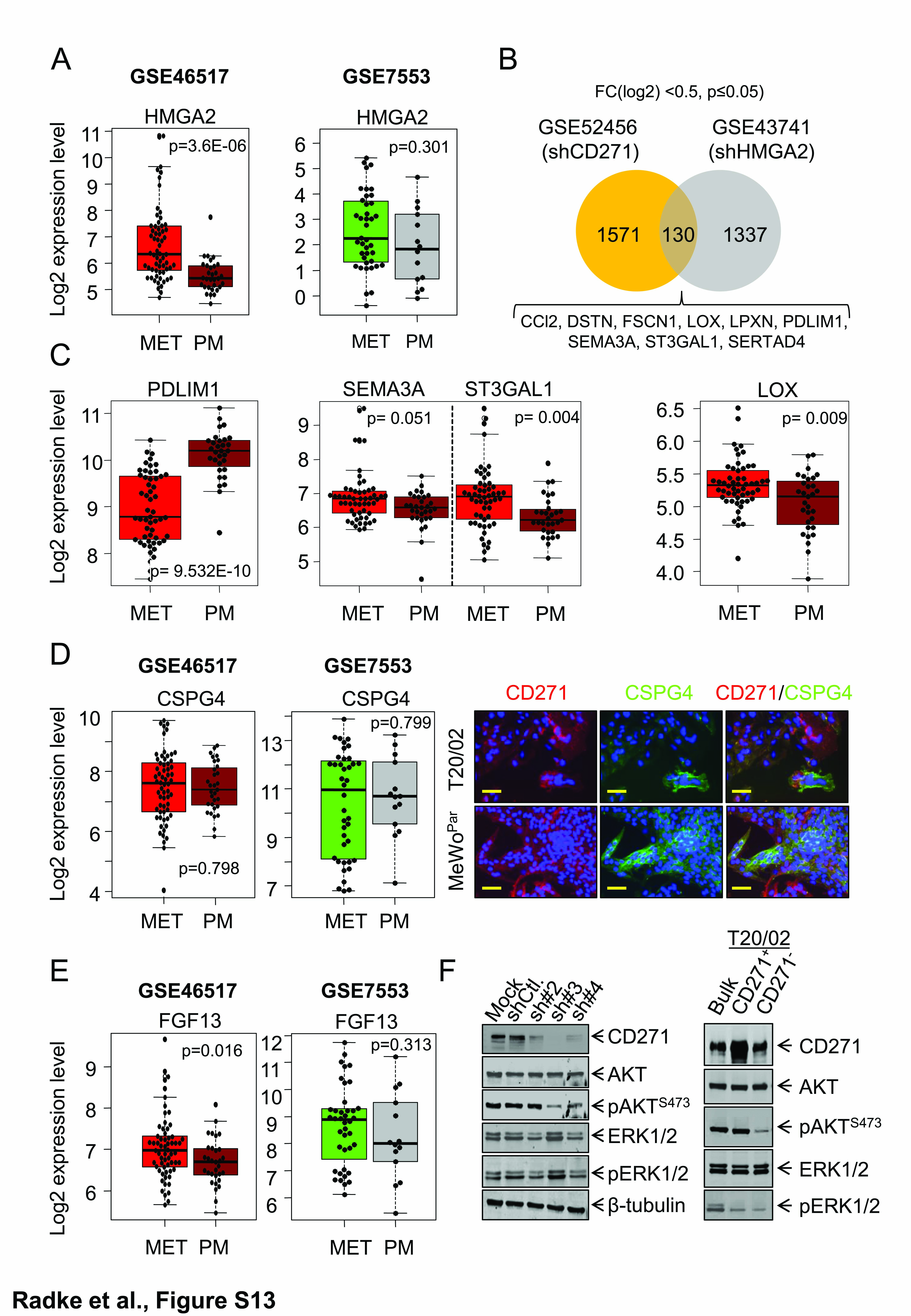


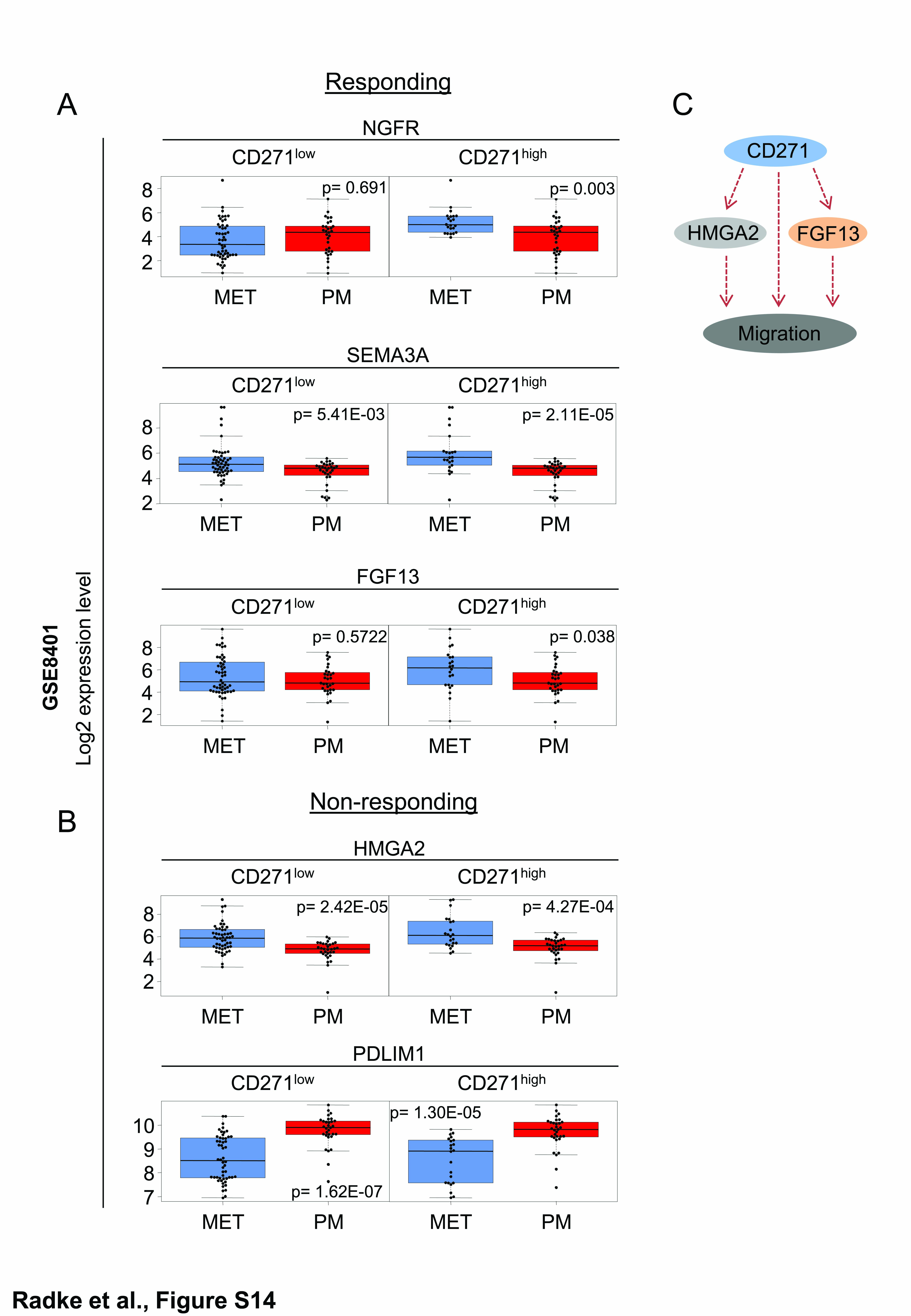


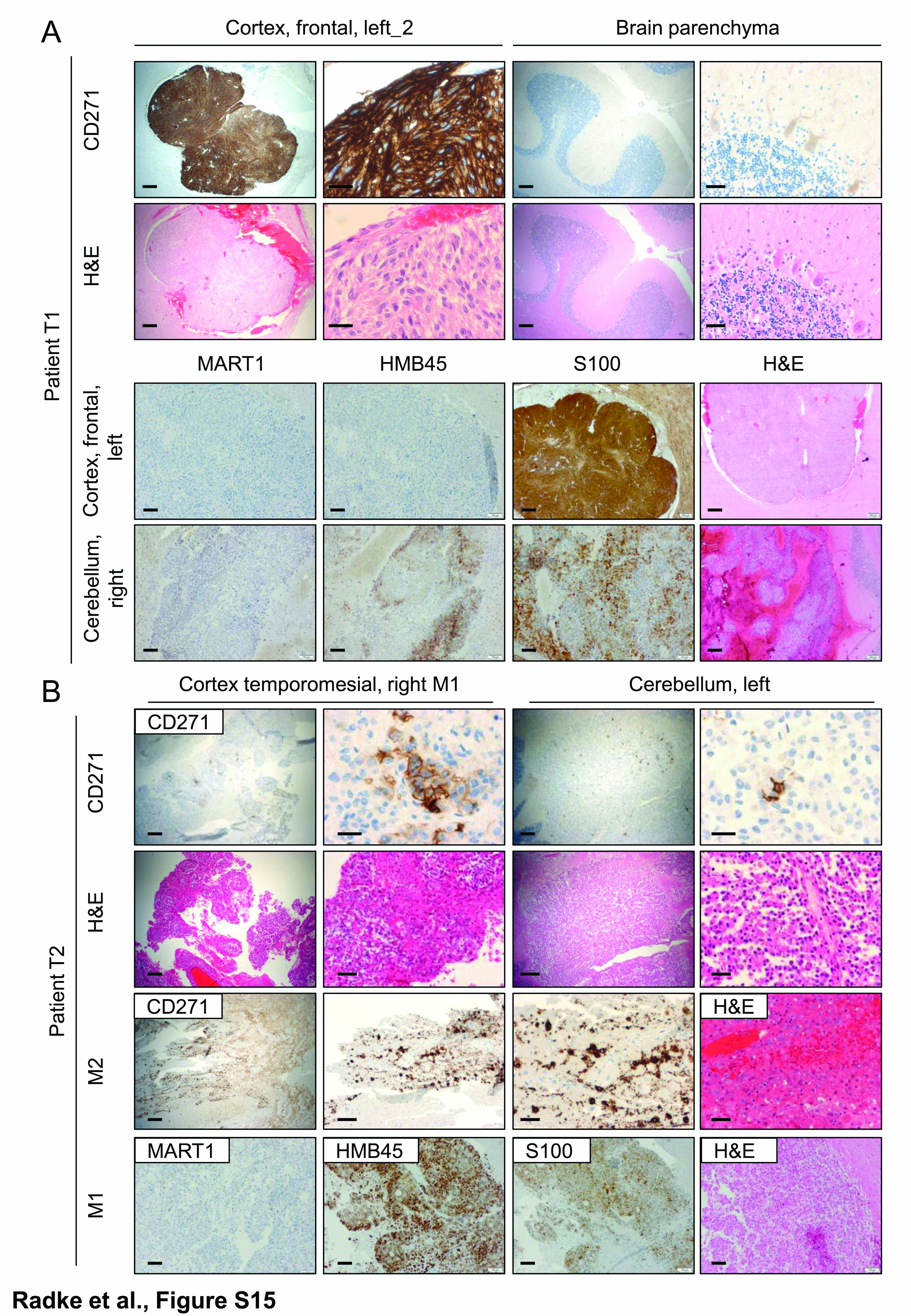


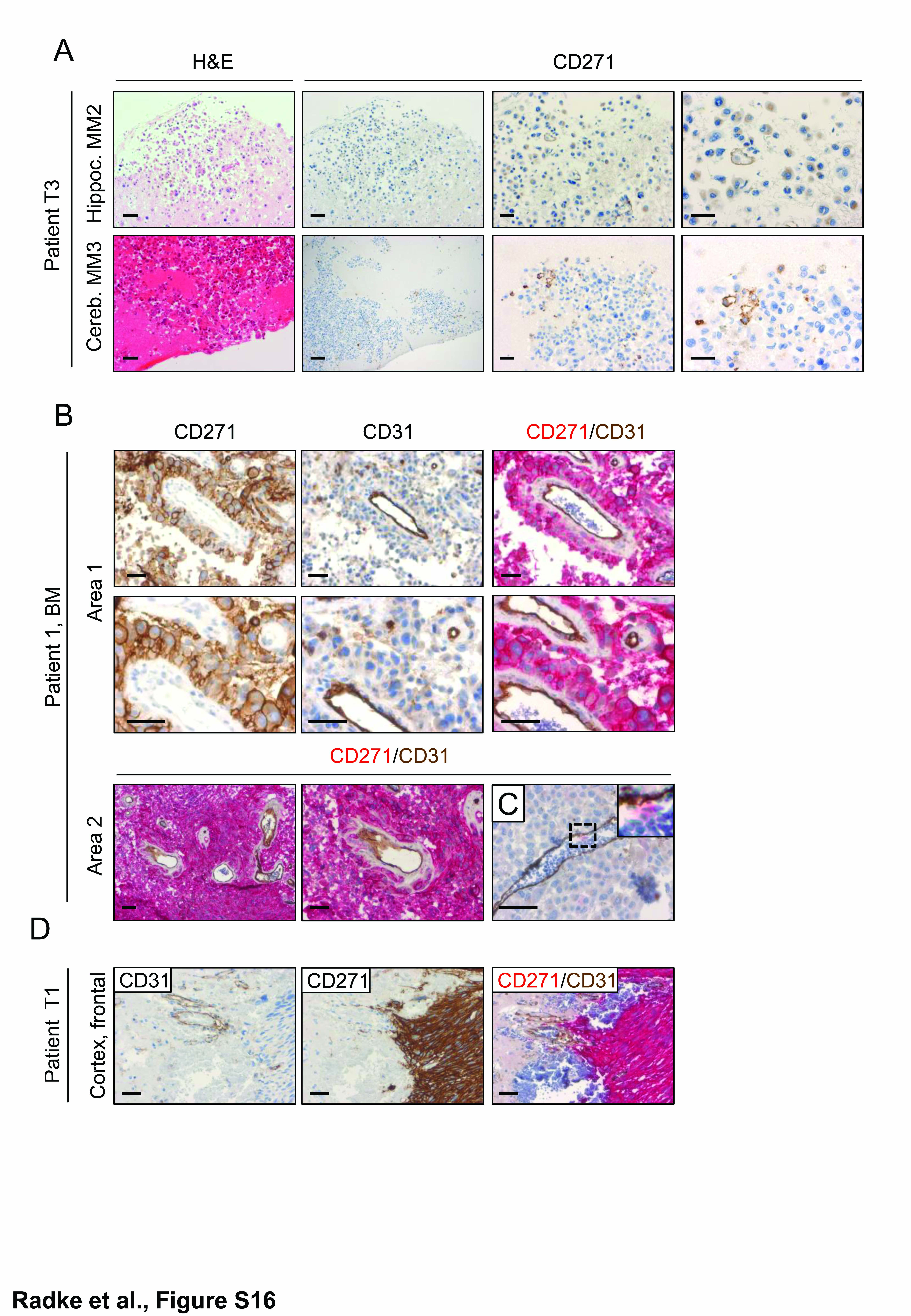


| **Pat.** | **CD271**  **(%)** | **MART1**  **(%)** | **Comments** | **MM type** | **BRAF**  **status** | **MET sites** | **Brain MET**  **sites** | **Add.**  **METs** | **Therapy** |
| --- | --- | --- | --- | --- | --- | --- | --- | --- | --- |
| **1** | 90 | 90 | Primary. skin | Desmoplastic | V600E | - | - | - | surgical treatment |
|  | 0 |  | - | Desmoplastic |  | Skin |  |  |  |
|  | 95 | 90 | - | Desmoplastic |  | Skin |  |  |  |
|  | 100 | 99 | - |  |  | Brain |  |  |  |
| **2** | 0.5 | NA | - | Lentigo maligna | wt | Skin | - | lung | DTIC. |
|  | 5 | 100 | nuclear staining |  |  | Brain |  |  | whole brain radiation |
| **3** | 10 | 50 | - | SSM | V600R | Soft tissue | - | lung | radiation. Ipilimumab. |
|  | 0 | 100 | - |  |  | Brain | cranial. frontal |  | Nivolumab |
| **4** | 0 | NA | - | No tumor | V600E | Lymph node | - | lung | DTIC. Vindesine. |
|  | 1 | 100 | - |  |  | Brain |  |  | Gemcitabine. Treosulfan |
| **5** | 10 | NA | - | SSM | wt | Lymph node | - | - | surgical treatment. |
|  | 2 | NA | - |  | wt | Brain |  |  | whole brain radiation |
| **6** | 20 | NA | - | SSM | wt | Lymph node | - | - | surgical treatment |
|  | 0.5 | NA | - |  | wt | Lymph node |  |  |  |
|  | 0.5 | NA | - |  | wt | Brain |  |  |  |
| **7** | 0.5 | NA | - | SSM | wt | Lymph node | - | - | surgical treatment. |
|  | 0.5 | NA | - |  |  | Brain |  |  | radiation. Pembrolizumab |
| **8** | 60 | NA | - | SSM | wt | Lymph node | - | - | unknown |
|  | 90 | NA | - |  |  | Brain | - | - |  |
| **9** | 90 | NA | nuclear staining | SSM | wt | Lymph node | - | - |  |
|  | 5 | NA |  |  |  | Brain | - | - |  |
| **10** | 100 | NA | Primary. eye | Uveal melanoma | wt | - | - | - | Gemcitabin/Treosulfan. |
|  | 0 | NA | - |  |  | HEP |  |  | anti-PD1 |
|  | 0 | NA | - |  |  | Brain |  |  |  |
| **11** | 0.5 | NA | - | Nodular | V600K | Skin | - | - | INF-a. radiation |
|  | 3 | 100 | - |  |  | Brain |  |  |  |
| **12** | 0.5 | NA | - | SSM | V600E | PER |  | - | whole brain radiation. |
|  | 0.5 | 100 | - |  |  | Brain | cerebellum |  | vemurafenib |
| **13** | NA | NA | - | SSM | V600E | Skin | - | - | surgical treatment |
|  | 3 | NA | - |  |  | Brain |  |  |  |
| **14** | 5 | NA | - | unspec. | NA | Brain |  |  |  |
| **15** | 0 | NA | - | unspec. | NA | Brain |  |  |  |
| **16** | 10 | 100 | - | unspec. | NA | Brain |  |  | surgical treatment. radiation |
| **17** | 0 | NA | - | unspec. | NA | Brain |  |  |  |
| **18** | 0.5 | NA | - | unspec. | NA | Brain |  |  |  |
| **19** | 0.5 | NA | - | unspec. | NA | Brain |  |  |  |
| **20** | 30 | NA | - | unspec. | NA | Peritoneum |  |  |  |

**Table S1**

**Table S2**

| **Patient** | **Metastatic regions** | **Extracranial METs** | **Therapy** | **CD271 expression** |
| --- | --- | --- | --- | --- |
| **1** | Cerebellum. right | NA | NA | high (100 %) |
|  | frontal cortex. left | NA | NA | high (100 %) |
| **2** | Temporomesial cortex. right | bone. kidney. heart. lung. peritoneum | multiple | Scattered, highly positive cells |
|  | Cerebellum. left |  |  | Scattered, highly positive cells |
|  | Temporomesial cortex. right |  |  | Scattered, highly positive cells |
| **3** | Frontal cortex. right | lung. liver. spleen | DTIC | scattered in micrometastases |
|  | Hippocampus. right |  |  | scattered in micrometastases |
|  | Cerebellum. right |  |  | scattered in micrometastases |
|  | Cerebellum. left |  |  | scattered in micrometastases |
| **4** | Cerebellum. right | NA | multiple | scattered, positive cells |
| **5** | Adenohypophysis | lymph node. liver | NA | median (40 - 50 %) |
| **6** | Falx cerebri | liver | NA | negativ, no brain MET |
|  | Eye. left |  | NA | negativ, no brain MET |
| **7** | Cortex. parietal. left | liver. bone. lung | NA | single positive cells |

**Table S3**

| **No.** | **Genes** | **Probe_ID_1** | **FC_log2_shHMGA2** | **p.value_shHMGA2** | **Probe_ID_2** | **FC_log2_shCD271** | **p.value_shCD271** |
| --- | --- | --- | --- | --- | --- | --- | --- |
| **1** | AMPH | 8139131 | -0.805 | 1.73E-02 | ILMN_1685834 | 0.145 | 3.63E-04 |
| **2** | AP1S2 | 8171481 | -0.267 | 3.34E-02 | ILMN_1766411 | 0.413 | 4.40E-03 |
| **3** | AP1S2 | 8171481 | -0.267 | 3.34E-02 | ILMN_2120273 | 0.411 | 9.34E-03 |
| **4** | ARHGDIB | 7961532 | -0.582 | 1.41E-02 | ILMN_1678143 | 0.129 | 3.56E-05 |
| **5** | ARMCX2 | 8174092 | -0.611 | 3.37E-02 | ILMN_1785170 | 0.317 | 2.57E-05 |
| **6** | BASP1 | 8104601 | -0.450 | 1.94E-02 | ILMN_1651826 | 0.066 | 2.14E-04 |
| **7** | BTG1 | 7965423 | -0.302 | 4.51E-02 | ILMN_1775743 | 0.491 | 5.29E-03 |
| **8** | CCL2 | 8006433 | -2.200 | 2.97E-03 | ILMN_1720048 | 0.373 | 8.84E-05 |
| **9** | CD55 | 7909332 | -0.299 | 1.05E-02 | ILMN_1800540 | 0.455 | 1.15E-02 |
| **10** | CD96 | 8081564 | -1.200 | 2.81E-02 | ILMN_1711573 | 0.240 | 1.57E-04 |
| **11** | CDH11 | 8001800 | -2.200 | 3.18E-03 | ILMN_1672611 | 0.289 | 1.58E-03 |
| **12** | CKAP4 | 7966035 | -0.265 | 1.37E-02 | ILMN_1790891 | 0.460 | 8.38E-02 |
| **13** | CMBL | 8110971 | -0.343 | 5.33E-04 | ILMN_1709634 | 0.146 | 9.49E-05 |
| **14** | CPD | 8006123 | -0.284 | 2.59E-03 | ILMN_1703074 | 0.131 | 5.74E-05 |
| **15** | CPT1A | 7949971 | -0.773 | 4.46E-02 | ILMN_1696316 | 0.451 | 9.23E-05 |
| **16** | CYB5R2 | 7946292 | -0.741 | 3.65E-02 | ILMN_1739576 | 0.044 | 5.14E-05 |
| **17** | CYP27A1 | 8048432 | -0.368 | 1.86E-02 | ILMN_1704985 | 0.064 | 7.55E-05 |
| **18** | DERA | 7954185 | -0.292 | 1.46E-02 | ILMN_1811551 | 0.395 | 8.02E-04 |
| **19** | DOCK10 | 8059413 | -0.521 | 2.97E-02 | ILMN_1702301 | 0.432 | 4.65E-03 |
| **20** | DSTN | 8061114 | -0.511 | 2.25E-03 | ILMN_1706426 | 0.471 | 1.97E-03 |
| **21** | DUSP6 | 7965335 | -0.848 | 4.81E-03 | ILMN_1677466 | 0.437 | 4.06E-04 |
| **22** | ECHDC2 | 7916229 | -0.652 | 5.94E-03 | ILMN_1671568 | 0.054 | 3.51E-05 |
| **23** | ECHDC3 | 7926152 | -0.289 | 3.70E-02 | ILMN_2072178 | 0.457 | 1.20E-03 |
| **24** | EFR3A | 8148333 | -0.377 | 1.44E-02 | ILMN_1664776 | 0.384 | 9.44E-05 |
| **25** | EGFR | 8132860 | -0.450 | 3.85E-02 | ILMN_1798975 | 0.428 | 1.10E-03 |
| **26** | EPDR1 | 8132369 | -0.478 | 4.08E-02 | ILMN_1675797 | 0.030 | 3.40E-05 |
| **27** | ETV4 | 8015806 | -0.481 | 3.75E-02 | ILMN_1753830 | 0.428 | 5.59E-02 |
| **28** | F2RL1 | 8106403 | -1.000 | 7.66E-03 | ILMN_2041190 | 0.380 | 8.17E-05 |
| **29** | FAM174B | 7991453 | -0.331 | 1.99E-02 | ILMN_1652797 | 0.485 | 4.51E-03 |
| **30** | FAM43A | 8084891 | -0.666 | 2.98E-02 | ILMN_1706015 | 0.156 | 4.81E-05 |
| **31** | FAM46B | 7914015 | -0.289 | 2.54E-02 | ILMN_1808011 | 0.381 | 9.41E-04 |
| **32** | FOXJ2 | 7953699 | -0.378 | 3.04E-02 | ILMN_1731648 | 0.468 | 4.52E-02 |
| **33** | FRMD5 | 7988260 | -0.473 | 4.54E-02 | ILMN_1727592 | 0.379 | 1.50E-04 |
| **34** | FSCN1 | 8131339 | -0.539 | 1.12E-02 | ILMN_1808707 | 0.152 | 2.27E-05 |
| **35** | FZD6 | 8147766 | -0.253 | 1.40E-02 | ILMN_1659297 | 0.444 | 3.74E-03 |
| **36** | FZD8 | 7933075 | -0.821 | 1.03E-02 | ILMN_1729368 | 0.307 | 2.82E-04 |
| **37** | GALC | 7980580 | -0.695 | 1.18E-02 | ILMN_1799744 | 0.222 | 8.38E-06 |
| **38** | GGCX | 8053429 | -0.293 | 1.02E-02 | ILMN_1758232 | 0.435 | 1.32E-03 |
| **39** | GLIS3 | 8159900 | -0.411 | 2.21E-03 | ILMN_2402600 | 0.277 | 4.13E-03 |
| **40** | GPX7 | 7901460 | -0.308 | 2.01E-02 | ILMN_1726030 | 0.120 | 3.31E-05 |
| **41** | GRIK2 | 8121225 | -0.321 | 2.83E-02 | ILMN_2396702 | 0.490 | 1.17E-03 |
| **42** | GRK5 | 7930894 | -0.454 | 1.98E-02 | ILMN_2096719 | 0.430 | 6.95E-03 |
| **43** | GULP1 | 8046906 | -0.470 | 4.99E-03 | ILMN_1802690 | 0.156 | 7.43E-05 |
| **44** | H2AFJ | 7954124 | -0.294 | 9.47E-04 | ILMN_1708728 | 0.437 | 1.65E-04 |
| **45** | HLA-A | 8179034 | -0.267 | 1.37E-02 | ILMN_1671054 | 0.325 | 5.34E-03 |
| **46** | HSD17B11 | 8101648 | -0.323 | 2.94E-03 | ILMN_1735367 | 0.479 | 6.54E-03 |
| **47** | ICA1 | 8138202 | -0.273 | 1.39E-02 | ILMN_1814787 | 0.198 | 5.89E-05 |
| **48** | IGFBP4 | 8007100 | -0.497 | 6.29E-03 | ILMN_1665865 | 0.171 | 1.51E-02 |
| **49** | IGFBP7 | 8100541 | -0.252 | 5.69E-03 | ILMN_2062468 | 0.102 | 8.77E-06 |
| **50** | IL1RAP | 8084794 | -0.487 | 4.82E-02 | ILMN_2357062 | 0.403 | 3.31E-04 |
| **51** | INPP1 | 8047069 | -0.396 | 3.20E-02 | ILMN_1667239 | 0.085 | 5.70E-05 |
| **52** | IRS2 | 7972745 | -0.278 | 1.27E-02 | ILMN_2083469 | 0.458 | 1.56E-02 |
| **53** | KAT2B | 8078227 | -0.492 | 1.02E-02 | ILMN_3243142 | 0.339 | 5.05E-03 |
| **54** | KCNS3 | 8040458 | -0.750 | 6.35E-03 | ILMN_2175112 | 0.104 | 8.51E-05 |
| **55** | LARP6 | 7990080 | -0.342 | 7.35E-03 | ILMN_1752810 | 0.109 | 5.26E-05 |
| **56** | LGALS8 | 7910706 | -0.641 | 6.98E-03 | ILMN_2353358 | 0.459 | 3.98E-03 |
| **57** | LHFPL2 | 8112803 | -0.261 | 3.35E-02 | ILMN_1747744 | 0.463 | 9.90E-03 |
| **58** | LMCD1 | 8077490 | -0.632 | 2.67E-02 | ILMN_1754969 | 0.380 | 7.76E-05 |
| **59** | LOX | 8113709 | -0.520 | 2.81E-02 | ILMN_1695880 | 0.175 | 2.05E-05 |
| **60** | LRRC6 | 8152962 | -0.455 | 2.98E-02 | ILMN_1739683 | 0.239 | 1.26E-05 |
| **61** | LSP1 | 7968126 | -0.427 | 4.45E-03 | ILMN_2355225 | 0.325 | 4.72E-03 |
| **62** | LY96 | 8146934 | -0.788 | 2.02E-02 | ILMN_1724533 | 0.245 | 1.17E-04 |
| **63** | MAP1B | 8106098 | -0.390 | 5.42E-03 | ILMN_2377900 | 0.182 | 2.19E-04 |
| **64** | MAT2B | 8109732 | -0.260 | 7.28E-03 | ILMN_1673960 | 0.387 | 7.39E-05 |
| **65** | MEF2C | 8113039 | -0.882 | 2.15E-03 | ILMN_1742544 | 0.293 | 1.54E-04 |
| **66** | MFGE8 | 7991234 | -0.420 | 4.85E-03 | ILMN_1756071 | 0.427 | 1.55E-01 |
| **67** | MGLL | 8090433 | -0.545 | 2.21E-02 | ILMN_1738589 | 0.080 | 1.65E-05 |
| **68** | MGST1 | 7954196 | -0.366 | 3.09E-02 | ILMN_2355168 | 0.192 | 1.56E-05 |
| **69** | MICB | 8177955 | -0.511 | 4.66E-02 | ILMN_1708006 | 0.390 | 3.61E-03 |
| **70** | MPP4 | 8058273 | -0.695 | 4.44E-02 | ILMN_1705579 | 0.403 | 3.09E-04 |
| **71** | MX1 | 8068713 | -0.458 | 4.56E-02 | ILMN_1662358 | 0.264 | 1.11E-03 |
| **72** | MYLK | 8090098 | -0.337 | 1.83E-02 | ILMN_1691476 | 0.270 | 1.35E-02 |
| **73** | NCAM2 | 8067985 | -0.979 | 6.04E-04 | ILMN_1749962 | 0.301 | 3.49E-04 |
| **74** | NEXN | 7902495 | -0.484 | 2.17E-03 | ILMN_1783276 | 0.494 | 1.06E-04 |
| **75** | OPN3 | 7925492 | -1.100 | 2.18E-02 | ILMN_1716988 | 0.350 | 1.93E-04 |
| **76** | P4HTM | 8079677 | -0.378 | 1.27E-02 | ILMN_3300313 | 0.340 | 3.96E-04 |
| **77** | PAPSS2 | 7928944 | -0.399 | 1.57E-02 | ILMN_2410929 | 0.262 | 6.52E-04 |
| **78** | PARP12 | 8143327 | -0.704 | 1.31E-02 | ILMN_1718558 | 0.297 | 6.44E-05 |
| **79** | PCSK5 | 8155898 | -0.272 | 4.81E-03 | ILMN_1767934 | 0.104 | 3.82E-05 |
| **80** | PDGFRL | 8144802 | -0.423 | 2.39E-02 | ILMN_1680339 | 0.292 | 3.41E-05 |
| **81** | PDLIM1 | 7935180 | -0.546 | 9.73E-04 | ILMN_1788955 | 0.367 | 3.27E-05 |
| **82** | PKIG | 8062852 | -0.352 | 4.22E-02 | ILMN_2406169 | 0.447 | 1.16E-03 |
| **83** | PLCE1 | 7929388 | -0.549 | 1.01E-02 | ILMN_1784447 | 0.236 | 5.01E-05 |
| **84** | PLK2 | 8112202 | -0.497 | 1.85E-02 | ILMN_1717706 | 0.228 | 2.36E-05 |
| **85** | PMP22 | 8012896 | -0.537 | 1.89E-03 | ILMN_1810864 | 0.147 | 8.07E-05 |
| **86** | PPCS | 7900546 | -0.265 | 4.21E-02 | ILMN_1776094 | 0.419 | 2.66E-03 |
| **87** | PPFIBP2 | 7938231 | -0.307 | 6.42E-03 | ILMN_1675656 | 0.346 | 5.21E-03 |
| **88** | PRRX1 | 7907222 | -0.494 | 2.25E-02 | ILMN_1739496 | 0.217 | 1.25E-04 |
| **89** | PRTFDC1 | 7932584 | -0.244 | 1.15E-02 | ILMN_1708516 | 0.167 | 6.30E-05 |
| **90** | PSD3 | 8149551 | -0.259 | 3.24E-02 | ILMN_1717477 | 0.430 | 6.41E-04 |
| **91** | PYGB | 8061447 | -0.572 | 1.54E-02 | ILMN_1778360 | 0.173 | 1.47E-03 |
| **92** | RASSF4 | 7927186 | -0.261 | 4.47E-02 | ILMN_1690566 | 0.214 | 4.94E-04 |
| **93** | RBMS2 | 7956261 | -0.348 | 4.81E-02 | ILMN_1755411 | 0.479 | 1.26E-03 |
| **94** | REEP3 | 7927786 | -0.344 | 1.97E-02 | ILMN_1722642 | 0.439 | 3.18E-02 |
| **95** | RFTN1 | 8085665 | -0.542 | 3.93E-02 | ILMN_1800787 | 0.239 | 9.50E-05 |
| **96** | RNF182 | 8116980 | -0.418 | 3.60E-03 | ILMN_3243112 | 0.263 | 6.94E-03 |
| **97** | RP9 | 8138950 | -0.244 | 4.98E-02 | ILMN_1687922 | 0.442 | 3.45E-02 |
| **98** | SASH1 | 8122637 | -0.393 | 4.51E-02 | ILMN_2185984 | 0.343 | 2.04E-03 |
| **99** | SCP2 | 7901513 | -0.635 | 4.24E-03 | ILMN_1694776 | 0.408 | 1.39E-03 |
| **100** | SEMA3A | 8140668 | -0.551 | 3.80E-02 | ILMN_1765641 | 0.280 | 6.73E-04 |
| **101** | SERPINE2 | 8059376 | -0.509 | 4.66E-02 | ILMN_1655595 | 0.285 | 3.38E-04 |
| **102** | SERTAD4 | 7909503 | -0.521 | 3.57E-03 | ILMN_1730794 | 0.279 | 2.35E-05 |
| **103** | SH3D19 | 8103166 | -0.311 | 2.12E-02 | ILMN_1653133 | 0.416 | 1.26E-02 |
| **104** | SH3PXD2A | 7936115 | -0.303 | 2.70E-02 | ILMN_1743103 | 0.027 | 4.63E-05 |
| **105** | SHROOM2 | 8165934 | -0.264 | 1.21E-02 | ILMN_1681777 | 0.066 | 9.99E-06 |
| **106** | SLC17A9 | 8064014 | -0.564 | 1.31E-02 | ILMN_1785405 | 0.467 | 5.21E-03 |
| **107** | SLC36A4 | 7950990 | -0.308 | 2.29E-02 | ILMN_2082324 | 0.278 | 1.70E-05 |
| **108** | SLC7A2 | 8144786 | -0.427 | 2.43E-02 | ILMN_1781400 | 0.459 | 2.07E-03 |
| **109** | SMAD3 | 7984364 | -0.390 | 1.03E-02 | ILMN_1682738 | 0.478 | 5.04E-02 |
| **110** | SNTB1 | 8152606 | -0.754 | 2.17E-03 | ILMN_1793410 | 0.176 | 3.17E-05 |
| **111** | SPTBN1 | 8041995 | -0.320 | 2.17E-02 | ILMN_1714228 | 0.468 | 3.50E-04 |
| **112** | ST3GAL1 | 8153021 | -0.690 | 3.58E-02 | ILMN_1683313 | 0.256 | 1.84E-04 |
| **113** | STARD3NL | 8132376 | -0.301 | 2.37E-02 | ILMN_2228873 | 0.182 | 3.47E-04 |
| **114** | SVIL | 7932796 | -0.536 | 8.73E-03 | ILMN_1671404 | 0.326 | 4.20E-05 |
| **115** | SYNM | 7986385 | -0.389 | 2.68E-03 | ILMN_1712075 | 0.058 | 1.85E-05 |
| **116** | TAX1BP1 | 8131975 | -0.265 | 5.03E-03 | ILMN_2374770 | 0.342 | 8.38E-04 |
| **117** | TBL1X | 8165911 | -0.267 | 4.31E-02 | ILMN_1744795 | 0.213 | 2.05E-04 |
| **118** | TCEA3 | 7913593 | -0.955 | 9.49E-03 | ILMN_1726928 | 0.221 | 1.26E-05 |
| **119** | TFPI2 | 8141016 | -0.347 | 2.03E-02 | ILMN_2068104 | 0.459 | 3.58E-04 |
| **120** | TIMP3 | 8072626 | -0.596 | 7.06E-03 | ILMN_1701461 | 0.177 | 3.49E-04 |
| **121** | TMEM106B | 8131539 | -0.338 | 2.81E-03 | ILMN_1726288 | 0.369 | 1.53E-03 |
| **122** | TNS3 | 8139500 | -0.676 | 5.04E-03 | ILMN_1667893 | 0.153 | 1.23E-05 |
| **123** | TRIM6 | 7938012 | -0.771 | 3.37E-02 | ILMN_1656910 | 0.167 | 2.40E-05 |
| **124** | UBE2L6 | 7948274 | -0.364 | 2.01E-02 | ILMN_1703108 | 0.404 | 7.11E-04 |
| **125** | UGP2 | 8042270 | -0.265 | 2.84E-02 | ILMN_2389155 | 0.480 | 7.16E-03 |
| **126** | VAMP8 | 8043197 | -0.648 | 1.32E-02 | ILMN_2190084 | 0.402 | 3.80E-03 |
| **127** | WDFY1 | 8059361 | -0.305 | 2.34E-02 | ILMN_1676448 | 0.372 | 8.86E-03 |
| **128** | WSB2 | 7966829 | -0.297 | 3.63E-02 | ILMN_1746664 | 0.483 | 1.00E-02 |
| **129** | WWC3 | 8165947 | -0.603 | 1.93E-02 | ILMN_1687592 | 0.236 | 5.29E-05 |
| **130** | ZFAND2A | 8137709 | -0.379 | 2.04E-02 | ILMN_1694671 | 0.373 | 2.79E-03 |
| **131** | ZNF671 | 8039680 | -0.455 | 1.48E-02 | ILMN_1713454 | 0.314 | 9.93E-04 |

**Table S4**

| **Gene** | **Entrez-ID** | **forward** | **reverse** |
| --- | --- | --- | --- |
| **FGF13** | NM_004114 | CACTCTGTTTAACCTCATCCC | CTTTGTTCAGACCCAGATACC |
| **CSPG4** | NM_001897 | CTGTATGAGCATGAGATGCC | CCAGAGACCTTTGTTCTTCC |
| **HMGA2** | NM_003483 | CAAGAGTCCCTCTAAAGCAGCTCA | TTCGGCAGACTCTTGTGAGGATGT |
| **AKT3** | NM_005465 | AAATAAACGCCTTGGTGGAGGACC | TTGTCCATGCAGTCCATACCATCC |
| **HPRT** | NM_000194 | GTTGTAGGATATGCCCTTGAC | GCCCAAAGGGAACTGATAGT |
